# A Many-Body Field Theory Approach to Stochastic Models in Population Biology

**DOI:** 10.1371/journal.pone.0006855

**Published:** 2009-09-01

**Authors:** Peter J. Dodd, Neil M. Ferguson

**Affiliations:** MRC Centre for Outbreak Analysis and Modelling, Department of Infectious Disease Epidemiology, Imperial College London, London, United Kingdom; University of East Piedmont, Italy

## Abstract

**Background:**

Many models used in theoretical ecology, or mathematical epidemiology are stochastic, and may also be spatially-explicit. Techniques from quantum field theory have been used before in reaction-diffusion systems, principally to investigate their critical behavior. Here we argue that they make many calculations easier and are a possible starting point for new approximations.

**Methodology:**

We review the many-body field formalism for Markov processes and illustrate how to apply it to a ‘Brownian bug’ population model, and to an epidemic model. We show how the master equation and the moment hierarchy can both be written in particularly compact forms. The introduction of functional methods allows the systematic computation of the effective action, which gives the dynamics of mean quantities. We obtain the 1-loop approximation to the effective action for general (space-) translation invariant systems, and thus approximations to the non-equilibrium dynamics of the mean fields.

**Conclusions:**

The master equations for spatial stochastic systems normally take a neater form in the many-body field formalism. One can write down the dynamics for generating functional of physically-relevant moments, equivalent to the whole moment hierarchy. The 1-loop dynamics of the mean fields are the same as those of a particular moment-closure.

## Introduction

The structure of biological populations in space, and the effects of random fluctuations, are well-established to have a significant influences on the dynamics of those populations. These range from qualitative differences, like the possibility of coexistence for competing organisms (e.g. [1]); through to acute quantitative differences, such as for epidemics where space provides the principle stratification of the population (e.g. [2], [3]). The problem of understanding these effects and their interplay is made more difficult by a lack of analytical machinery, which leads to a reliance on extensive numerical simulation. Even with modern computers, this can make certain tasks requiring very many realizations too slow to be useful in situations where rapid answers are required (e.g. real-time estimation of model parameters during epidemics).

Beyond mean field theory, the main approach which has been brought to bear is the technique of so-called moment-closure. If one examines the dynamics of the mean fields in such systems, one typically finds that they include a dependence on the second moments. The dynamics of the second moments include a dependence on the third, and so on. In this way, one obtains a hierarchy of equations governing the evolution of the moments, which can be thought of as equivalent to the full stochastic system. Moment-closure means truncating this hierarchy (almost always at the second moment) by positing that the moments at a certain order are some function of the lower order moments. This is an uncontrolled approximation, and one drawback is that the choice of closure function must be guided by experience, or by a posteriori comparison with simulations.

In [4] and [5], it was first noted that certain stochastic systems on lattices can be rewritten in the language of quantum field theory (QFT). Since then, this rephrasing has mainly been used to obtain critical exponents for percolation-like systems, via renormalization group techniques (see e.g. [7]). Here, we will argue that for the kinds of model studied in population biology and epidemiology, this field theoretic description is notationally neater and more manageable than standard methods, in often replacing sets of equations with single equations with the same content. The master equation (Kolmogorov forward equation) takes the form of a Schrödinger equation in imaginary time. A single Hamiltonian sums up the dynamics compactly, even when births and deaths allow the population size to change; and the moment hierarchy is summarized in a single equation for the dynamics of a moment-generating functional.

The introduction of coherent state path integrals allows access to much of the functional machinery used in QFT, for example diagrammatic perturbation theory. We will concentrate on the effective action. Functional differentiation of the effective action yields the exact dynamics of the mean fields, including all stochastic and nonlinear effects. There is a systematic procedure for iteratively computing the effective action, known as the loop expansion. The term loop refers to the diagrams involved in calculating each iteration. We shall not introduce diagrammatic technology, but calculate the 1-loop term for the general case and corresponding dynamics for two specific models.

In the next section, we will describe the two models we study, and then use them to introduce field theoretic language. We explain how the spatial distribution functions fit naturally into this picture. We go on to explain the path integral representation, the loop expansion of the effective action, and establish a general result for computing the effective action.We then write down the actions for our models and compare the 1-loop dynamics with the usual moment-closure approaches, before summarizing.

## Methods

### 1 Creation and annihilation operators

Reference [8] considers a population of ‘bugs’ which undergo Brownian motion with diffusion coefficient κ, and spontaneously give birth by binary fission as a Poisson process with rate λ. The *i*th bug also has a hazard of dying which is the sum of a background rate μ and the quantity 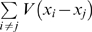. *V* is a competition kernel which enhances the chances of a bug dying if it is close to other bugs, and models something like a competition for resources. The model is therefore one of spatial, stochastic logistic growth, with diffusion added.

Consider first a non-spatial version for simplicity. The master equation for the evolution of the probabilities is
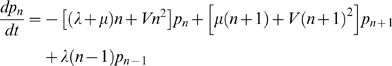
(0)This equation represents the flux of probability between states at rates defined by the model. We will instead represent the probabilistic state of the system as a vector
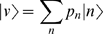
using Dirac notation for vectors (“kets” |α〉), their duals (“bras” 〈α|), and their inner products (“brackets” 〈α|β〉).

We introduce annihilation and creation operators *a* and 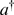 respectively, satisfying the commutation relation
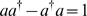
(1)and build the space from basis vectors of the form 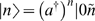 (see Box S1). The reference state |0〉 has the property *a*|0〉 = 0 (whence ‘annihilation’ operator), and also that 〈0|0〉 = 1 in the inner-product of the space. The idea is that 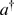 ‘creates’ bugs, so the vector 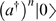 represents the probability distribution where there are definitely *n* bugs; 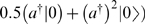 corresponds with a distribution where there are 1 or 2 bugs present each with probability 0.5; and so on. Thus, we can write our state as
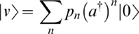
(2)The commutation relation Eqn.1 for *a* and 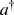 implies that 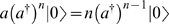, and thus 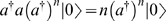. The operator 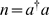 therefore counts the bugs in a definite state, and is called the number operator.

Also of particular importance is the concept of normal ordering (denoted :(…):), which means inside the colons, moving operators with daggers to the left of all those without daggers. For example, 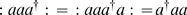, and so on. We will also introduce the reference state |〉 = exp(*a* ^+^)|0〉. This coherent state (see Box S3) is useful because a state corresponding to a probability distribution satisfies the normalization condition 〈|*v*〉 = 1, which is equivalent to 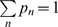. With this notation, expectations can be written in a quantum-like fashion (e.g. the expected number of bugs in state |*v*〉 is *n* = 〈|*n*|*v*〉). The master equation is linear, and can be written in a Schrödinger-type form
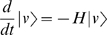
(3)The linear operator, *H*, which generates the time evolution is called the Hamiltonian. It is easy to check that for our non-spatial logistic growth model Eqn. [0], one can write the Hamiltonian in terms of creation and annihilation operators as
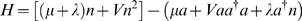
(4)The term in square brackets consists only of number-operators and corresponds to the first term in Eqn.[0]. The term μ*a* corresponds to the death term μ*(n+1)p_n+1_* of Eqn.[0], with the annihilation operator *a* acting to destroy a bug: *a*|*n*〉 = *n*|*n−1*〉. The actions seem to “go the other way” to the corresponding terms in Eqn. [0] because the operators act on the vectors as opposed to the coefficients.

The gain in simplicity is for the spatial case. Because the number of bugs can vary, one needs many marginal probability distributions 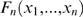, conditional on there being *n* bugs. These must be symmetrized because the bugs are identical, and are normalized so that integrating out all the spatial arguments of each one, and then summing over *n* gives 1:
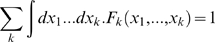
The expectations of observable quantities are similarly sums over terms in each *n*-bug sector, and the master equation must be specified for each *n* separately and includes clumsy symmetrization operations (see [8]).

On each count, the field theory version is more succinct. As above, one introduces local creation and annihilation operators 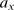 and 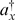, this time such that
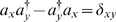
(5)where 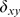 is a Dirac delta function (see Box S2). (We work in units where the area of the spatial domain has value 1. Area factors can be reintroduced by dimensional analysis.) Our state vector will be
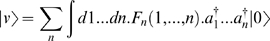
(6)which is automatically symmetric.(Here and elsewhere, we avoid repeated subscripts by writing *k* for 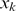.) With the reference state 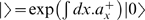, the normalization condition again reads 〈|*v*〉 = 1. Expectation values are given as above, e.g. the expected local number density is 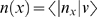 (for local number operator 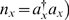; and the master equation again becomes Eqn.3, this time with Hamiltonian
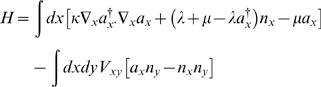
(7)This is simpler because one does not have to specify the dynamics for each possible number of bugs separately. Each term in the Hamiltonian has a clear interpretation, and with practice, a Hamiltonian can be written down straight from the verbal description of the possible transitions involved in the model.

The term moment hierarchy refers to reduced spatial distribution functions 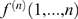. E.g., 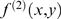 is the probability density of finding a bug at *x* and another at *y* at time *t*, and is given in terms of a sum over the appropriately symmetrized marginals:
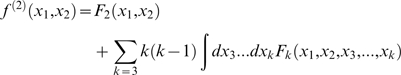



That is, one calculates the probability of finding a bug at *x* and at *y* given there are *n* bugs by integrating out all but two spatial variables in every possible way, and then sums over all the possible numbers of bugs *n*. In the many-body field-theory (MBFT) formalism we have
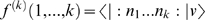
(8)where the colons denote normal ordering, as described above. This automatically takes care of the self-correlation terms one would otherwise have (see e.g. [6]). For example, if 
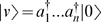
,
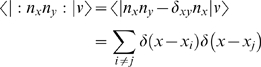
which avoids the singular ‘self’-terms with *i* = *j*.

If one introduces a generating functional, *Z*, for these distributions
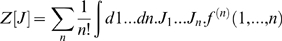
(9)such that the *n*-th distribution can be recovered by functionally differentiating *n* times and setting *J* = 0 (see Box S4), then Eqn.8 and the master equation imply that, for a Hamiltonian expressed in normal ordered terms, 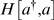,
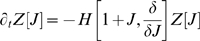
(10)This equation is equivalent to the entire moment hierarchy, and provides a convenient recipe for calculating the dynamics of a given moment. This is to be compared with taking the master equation for each of the 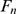, and using this together with the definition of 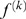 in terms of symmetrized marginals of the 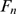 to obtain its dynamics. It is analogous to the recipe for generating function dynamics of non-spatial continuous time Markov processes described in [9].

The other model we will consider is a susceptible-infected-recovered (SIR) epidemic model on a population of Brownian bugs. The diffusion coefficient will again be κ, the recovery rate ν, and the rate at which infection takes place will be modulated by a spatial kernel *V*, which implies that proximity of a susceptible to infecteds increases the likelihood of infection. Now we have 3 types of creation and annihilation operator, each commuting with the other: *a*, *b* and *c* for susceptible, infected and recovered respectively (referred to in vector notation when convenient). The Hamiltonian reads
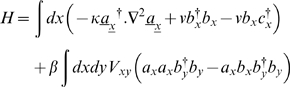
(11)The non-spatial version is just the usual stochastic SIR model. The Hamiltonian is
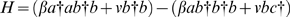
(12)


### 1.1 Example: calculation of moment dynamics

In this section we provide a worked example of the application of Eqn.[10] in obtaining moment-dynamics. Consider a spatial SIR model without diffusion. Using the Hamiltonian of Eqn.[11] with κ = 0, and writing *A,B* and *C* for the fields corresponding to susceptible, infected and recovered individuals respectively, Eqn.[10] becomes:
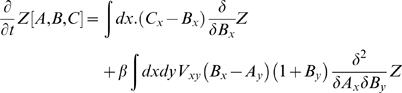
Recall that *Z* is a generating functional for the physical moments (Eqn [9]): setting *A = B = C = 0* makes both sides zero, as it should. To obtain the dynamics of the first moments, we must differentiate once with respect to the fields, before setting the fields equal to zero. The manipulations in functionally differentiating integrals with respect to the field *A*, e.g., are formally analogous to partial differetiation of sums over indexed variables, with the spatial label playing the role of an index. Bearing in mind that we will set the fields zero, only terms which do not contain a factor of *A, B*, or *C* after differentiation will survive. Thus:
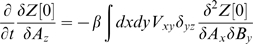
or in terms of the moments:
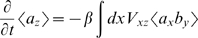



To calculate the dynamics of the second moments, one must differentiate twice before setting the fields equal to zero. For example, in calculating 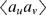, the non-zero terms read:
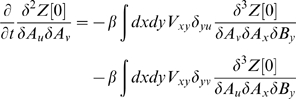
or for the moments:
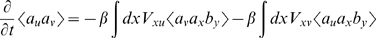
Other terms are obtained similarly, using a procedure which could be easily automated with computer algebra software.

### 2 Functional methods

Path integrals were conceived of by Feynman in the context of quantum mechanics ([10]), and have since proved especially useful for QFT and statistical mechanics (see Box S5 and e.g., [11] and [12]). They are founded on splitting the evolution into many short time intervals, and inserting a particular resolution of the identity operator at each step. In the stochastic context, this amounts to the use of the Chapman-Kolmogorov equation on many time-slices. The result is an integral over functions, which must be treated as a limit of an *N*-fold integral over a discretized version of the integrand, as *N*→∞. A path integral can be thought of as giving the probability of going from one state to another as weighted sum of all the possible histories between those states.

The idea is that if we treat φ(*x*,*t*) as an infinite-dimensional vector, indexed by *x* and *t*, functionals (with square brackets) are the analogues of real-valued functions; functional differentiation is the analogue partial differentiation; and functional integration the analogue of volume integration over the vector space.

Coherent states correspond with spatially-varying Poisson distributions, and are defined by
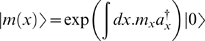
(13)(e.g. our reference state |〉 is a special case). They are eigen-states (i.e. eigenvectors) of the annihilation operator. We follow the coherent state conventions of [6] and [7], and ‘shift-trick’ of [7] (see the supplementary material S1 for more details). Then the expectation of a normal-ordered operator *X* for example (for simplicity, referring to only one time), given an initial coherent state 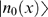, has the functional representation
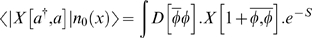
(14)where the coherent state path integral for a time interval [0,*t*] is a sum over field histories which have 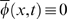 and 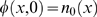. The quantity *S* is the action, given by
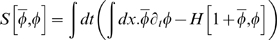
(15)if 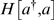 is the Hamiltonian written in normal ordered form. In the functional formalism, the action plays an analogous role to the Hamiltonian, in that it encodes the dynamics of the system. Note that coherent state path integrals are somewhat different to Feynman path integrals: see [11] for a comparison, and [6] for more details on the coherent state version.

Normal ordering is naturally built into path integrals, so that, for example, we have
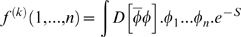
(16)and therefore
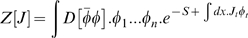
(17)It is Eqn.17 and its generalizations to unequal times and source terms for 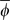 which serve as the starting point for diagrammatic perturbation methods. Perturbing an action by a term −εΔ*S* means that *Z* changes to exp(εΔ*S*[δ/δ*J*]).*Z*, and terms at each order in ε can be represented, organised and manipulated as diagrams. We will not pursue this here.

Below, we will see that the action, *S*, can be considered the zero-loop approximation to the effective action. Varying this with respect to the fields (i.e. differentiating with respect to the fields) provides the most basic approximation to the dynamics, usually resembling the mean-field equations.

### 3 The Effective Action

Hereon, we will frequently neglect writing out integrals explicitly, and use *J* and φ to schematically refer to both barred and un-barred quantities. *Z* will now denote the generating functional as above, but with *J*φ now representing 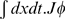, or rather a sum of terms like this for all fields. *W* = log*Z* is the cumulant generating functional. We define a quantity Γ by the Legendre transform:
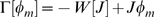
(18)

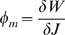
(19)Γ is the effective action and has the property that
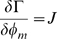
(20)


The significance of this for us is that at *J* = 0, the effective action yields the exact equations of motion for the mean fields, including all stochastic corrections (see Eqn.19 and Eqn.20). Parenthetically, we note that both *W* and Γ have interpretations as sums over subsets of the diagrams involved in *Z*.

### 4 Calculating the effective action

There is a standard method for iteratively calculating the effective action. Let us introduce the difference between the action and the effective action Δ = Γ−*S*, and a counting parameter 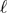. Using the definition of Γ in Eqn.19, the expression for *J* in Eqn.20 and the functional integral expression for *W*, one can establish after a shift and rescaling of integration variables (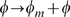 and then 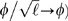) that
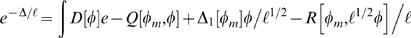
(21)(For more details see the supplementary material S1 or a textbook like [13].) Here *Q* is the quadratic part of the action, expanded as a functional taylor series around 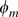; *R* is the sum of the third and higher order terms in the action's taylor series around 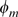; and an integer subscript *k* means the *k*th functional derivative.

This can be solved recursively and results in a series for Γ of the form
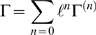
(22)Although it would appear from Eqn.21 that there should be non-integer powers of 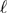 in Eqn.22, this is not the case because only even moments of gaussian integrals are not zero. It turns out that the power of 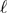 corresponds to the number of loops in the diagrams associated with the shifted theory (*Q* as the action), and that the number of loops roughly corresponds to the degree of calculational complexity. Thus one hopes that the simplest (e.g. the 1-loop term) terms already contain the bulk of the significant information. In the quantum case 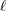 is the fundamental constant 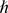; for us 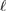 is the inverse area of the system.

From Eqn.21 and Eqn.22 the 1-loop expression reads
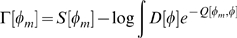
(23)Recall that *Q* is the quadratic part of the action expanded around 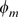, and the integral is over fields which are zero at 0 and *t*.

Very often in the field theory literature, this is restricted to the stationary, translation invariant case (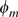 a constant). The result is the so-called effective potential, which provides information about equilibrium states. We will later restrict to the translation invariant case, in common with the biological literature; but maintain the time-dependence of 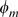 so as to gain information about the non-equilibrium dynamics. To do this, we need a result on Gaussian many-body path integrals from the next section.

### 5 General fluctuation integral

In order to calculate the 1-loop effective action then (and indeed, higher-loop terms), one needs to be able to calculate the integral
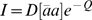
(24)where the integral has zero limits and
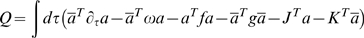
(25)The quadratic parts here are the quadratic parts of the Taylor-expanded action, and we use matrix notation to deal with the case of an arbitrary number of fields. In the supplementary material S1, we extend the result of [14] to this multi-field, non-Hermitian case with sources - necessary for stochastic systems - closely following their methods. Up to an irrelevant constant factor, we find
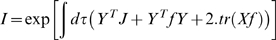
(26)where, with trivial initial conditions, *X* and *Y* satisfy
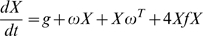
(27)

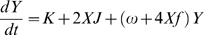
(28)This means that up to 1-loop, the effective action is simply
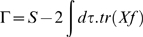
(29)where *X* satisfies the differential equation of Eqn.27. The equations of motion obtained by varying this should be such that barred fields are identically zero. This must be the case due to the reference state, and simplifies the resulting equations significantly (e.g., in varying the 1-loop correction, only *tr*(*X*δ*f*) survives). However, it is only legitimate to set the barred fields to zero *after* variation. We note in passing that constant multiples of *Q* yield the same result, because additive constants are irrelevant to Γ.

### 6 Eyink's variational principle

In [15], Eyink showed that Eqn.[3] can be obtained from varying the quantity
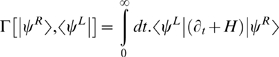
subject to the constraint 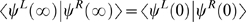. This is analogous to the Dirac action in quantum mechanics, and for stationary situations, reduces to the Rayleigh-Ritz variational principle which is known in a wider context. He went on to show that this is a representation of the effective action, and that using an Ansatz for |ψ*^R^*〉 which is parametrized in terms of its moments, *m_i_*, the variation yields the dynamics:
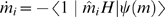
(29.5)where 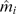 is the operator giving the moment: 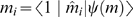. This formalism has already been used at least once to derive a novel approximation in a biological context [16].

The meaning of this for the population biologist is that if one chooses the probability distribution to from some family which can be parametrized by some of its moments (like the Gaussian distribution, e.g.), the dynamics of those moments is given by the moment-closure associated with that distribution. So if one has a reason (such as numerical experiment) for suspecting that the distribution of interest should look Gaussian, zero-central-moment is an appropriate closure, and can be thought of as the dynamics on this family derived from the effective action. Similarly, closures which have been named due to their form (e.g. the “Poisson” closure), do actually derive from the corresponding distribution in this sense. This link provides an alternative motivation for moment-closures, and a less ah hoc way to make an appropriate choice.

## Results

In this section, we apply the methods outlined above to the example models of the previous section. For more detailed versions of the calculations in this section, see the supplementary material S1. In each case, we are interested in the dynamics of the mean fields at 1-loop. The equations for the barred fields are omitted as they admit trivial solutions (i.e. barred fields have been set to zero after variation).

### 1 Non-spatial versions

For the simple bug model, it will be convenient to introduce the quantity γ = λ−μ. The action for the system becomes
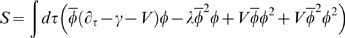
(30)This yields at 1-loop:
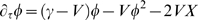


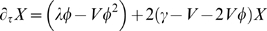
The zero-loop equation is simply the equation for 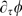 with *X*≡0. This is of interest because it differs from the naive mean-field equations by the presence of the *V*φ term.

The non-spatial SIR model has the action
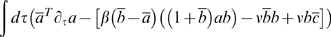
This time the zero-loop equations are the same as the mean-field equations, and therefore the same as the differential equations of the usual deterministic SIR model. The 1-loop corrections are obtained following the above recipe, and require appending some differential equations linear in *X*. Only 3 of the *X* differential equations are relevant to the correction (see supplementary material S1).

### 2 Spatial versions

In compact notation, the actions for the bug population model and the epidemic model are
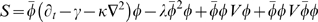
(31)and
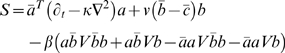
(32)respectively. To clarify, we have omitted all integrals. Where there are double integrals over space, quantities to the left of the *V* carry one argument, and quantities to the right the other. So, for example: 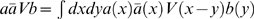 etc.

For these spatial cases, we consider our bugs as residing on a torus. That is, we assume that ‘space’ is a square with length *L*, and with pairwise identification of opposite edges. All functions on this space are periodic, and so it is natural to represent things in terms of their Fourier modes, i.e.
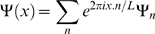
(33)where 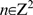. We will (as is usual) assume a spatially homogeneous (translation-invariant) initial state. In this case, the mean fields are constant in space φ(*x*) =  φ, and the quadratic part of the action can be written as a sum of Fourier modes
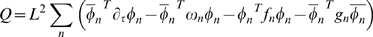
(34)which do not interact. Up to 1-loop therefore, the effective action is given by
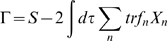


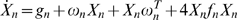
(35)where in our case the quadratic term in 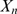 is not present after setting barred fields to zero. This means the 1-loop dynamics are given by
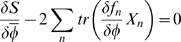


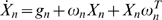
(36)where these are both at 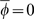, and the *S*-term gives the mean-field dynamics.

Although this infinite set of equations may look fearsome, one can convert it back into real space, where it has the same structure as the equations one obtains with moment-closure methods. Indeed they are the same as the closure which results from setting the third cumulant density identically to zero (zero third central moment closure).

In the bug model for example, both the zero third central moment and the 1-loop dynamics have the form (see supplementary material S1):
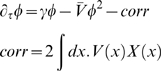
with
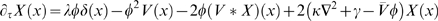
where 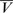 is the integral of *V* over all space and V*X is the convolution of *V* with *X*.

### 3 Perturbative expansion around the mean-field solution

In this section we derive a novel approximation to the non-spatial SIR model, as an illustration of calculations using the formalism. The probability of being in a state 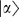 at a time *t,* having started in 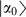 at time 0 is:
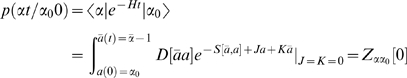
One can expand this path integral around the stationary path for the action (the mean-field dynamics) to give
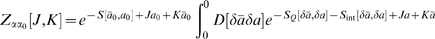
where
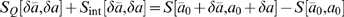

*S_Q_* being the quadratic part and *S_int_* the rest. The integral term contains all information about the (non-Gaussian) fluctuations. This means, for example, that
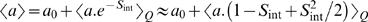
where the expection with respect to the quadratic part of the action can be computed (perturbatively), since the Gaussian path integral is tractable. The approximate equality results from a naïve, but systematically improvable Taylor expansion of the exponential of the interaction part of the action. This last can be computed using the results above on the general fluctuation integral. The barred mean-field solutions are identically zero, so *f = *0, in the notation used above reducing the fluctuation integral to
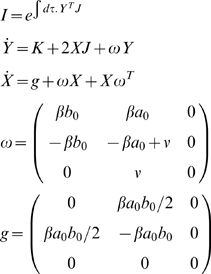
Differentiating the expression for *I* shows that only moment of even numbers of fields are non-zero, and that these are equal to sum over all possible products of second moments involving these fields. This is a special case of a more general result known as Wick's theorem (see [10]). It is also easy to see that moments are only non-zero if there are more unbarred fields than barred. It remains to calculate the derivatives of *Y* with respect to *J* and *K*. This can be done be differentiating the differential equations for *Y* and *X* and then solving them, and one obtains:
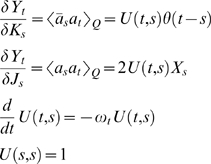
Where θ is equal to 1 if *t>s* and zero otherwise. The value θ(0) can be justified by careful consideration of the discretized version of the differential equation for *Y*, or by computation in the operator formalism. This means that many terms are equal to zero, and the approximation becomes:
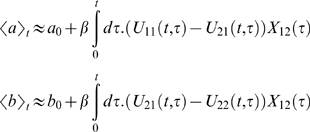
Despite the fact that this approximation is poorly-motivated, its performance is better than that of mean-field theory, though inferior to third-cumulant-zero moment-closure. Comparisons were made for population sizes of the order hundreds, where deviations between the stochastic model and its mean field description would be fairly pronounced. The Taylor-expansion approximation shared the problems of moment closure at small numbers of initial infected individuals, but was less stable with typically larger deviations from the mean of the stochastic model. See Figure S1 for representative output.

### 4 Comparisons

Some comments are in order. The 1-loop equations of motion have turned out to be the same as those from taking the normal-ordered third cumulant to be zero. This is because the 1-loop procedure is essentially a WKB approximation, and Gaussians have zero third cumulants. In the spatial case, taking the normal ordered third cumulant to be zero is the physically natural thing to do. In the non-spatial cases, it is the non-normal ordered moments which are meaningful. Thus in the non-spatial cases, setting the normal-ordered third cumulant to zero is distinct from the usual zero third central moment closure, which is defined with non-normal ordered moments. As mentioned above, the equations for the non-spatial bugs model differ at zero-loop from naive mean-field theory, and in form from those of the spatial bugs model. The extra term is from normal ordering the Hamiltonian, and the latter difference is down to the fact that one does not need to worry about avoiding self-interactions in the non-spatial case.

It should be noted that in these non-spatial cases where the 1-loop approximation differs from the usual central moment closures, the performance of the 1-loop approximation in describing the mean dynamics of the stochastic model is at least comparable with that of the moment-closure approximation; suffering from the same sorts of problems, for example when initial numbers of infecteds are very small in the SIR case. It also should be noted that the extra term in zero-loop approximation to the non-spatial bug population model significantly improves its performance compared with naive mean-field equations.

## Discussion

The main aim of this paper was to introduce field-theory as a natural language for describing spatial stochastic models in population biology and epidemiology. We feel the Hamiltonian, which describes the system dynamics, is usually simpler in form than a traditional master equation, especially when the total population size is allowed to vary due to the unified treatment of populations with different sizes. Moreover, with practice, it is straightforward to write down a Hamiltonian from a verbal description of the dynamical rules. For example, the laws governing the spatial ‘bug’ population model are fairly easily explained, and yet the master equation in the traditional form of [8] is formidable in appearance, and difficult to interpret and manipulate. More manageable notation should allow and encourage analytical manipulation and investigation of models - and even formulation of models - where calculational difficulty has previously got in the way. Table 1 provides some examples of rules for transition rates common in biological models, and their corresponding contributions to a many-body Hamiltonian describing the system.

**Table 1:** A recipe-book for constructing Hamiltonians from transition rates.

	Transition	Contribution to H
**One-body terms**	diffusion of *a*, rate κ	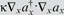
	death of *a*, rate μ	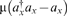
	change (*b*→*c*), rate ν	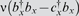
**Two-body terms**	competition, kernel *V*	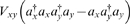
	infection,(*a*→*b*)	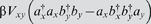
	birth, dispersal kernel *V*	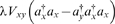

One-body terms are integrated over *x* and two-body terms are integrated over both *x* and *y*. The positive terms correspond to the efflux of probability from a configuration state, and the negative terms effect the flow of probability into a new state.

Spatial correlations fit naturally into this framework in terms of normal-ordered moments, and we showed Eqn.10 provides a particularly concise description of the dynamics of the moment hierarchy. If interested in moment-closure, the extra ‘δ’ terms which must be carefully included in the closure relation of some approaches (see e.g. [2]) are automatically incorporated in the dynamics, and one only needs to set the third derivative of log*Z* to zero. (A caveat here is that the non-spatial analogue of Eqn.10 still deals with normal-ordered correlations.).

The approach of Ovaskainen and Cornell [17] provides one of the most successful new approaches to stochasticity in spatial population dynamics of recent years. They develop a perturbation theory in the inverse area of the system (the loop parameter is also proportional to the inverse area as noted above) with a superior performance to 1-loop/zero third cumulant moment-closure. We note however that some of their calculations would be simpler with the methods presented here. Their transition between moments *G* and G^*^ (their Equations 4–6) is precisely a shift to normal-ordered correlators which we have denoted *f*. Such a shift is not necessary above as we work with the *f* from the start. In their supplementary material S1, they calculate various moment dynamics and then transform to Fourier space. This could be avoided by using the above methods by writing *H* in terms of Fourier modes, and then using Equation.10 to calculate the dynamics of the normal-ordered correlators in Fourier space directly.

Apart from notational elegance and its concomitants, the other reason for turning to field-theoretic descriptions is that it can provides access to new tools. We chose to explore the use of effective action, and extended the result of [14] to the form needed for general stochastic systems, allowing calculation of the non-equilibrium 1-loop dynamics. In fact this yielded nothing new for the spatial models, in the sense that it returned the same equations as third cumulant zero moment-closure, albeit via an alternative calculation.

We note in passing that Eyink's variational principle provides a different understanding of moment closures as resulting from constrained variation of the effective action using a particular form of Ansatz. It also provides a method to develop more effective moment-closures by examining the form of the correlations or other moments generated by numerical experiment, and choosing a parsimonious Ansatz which describes their different forms through time.

Our perturbation approximation was inferior to moment-closure, unsurprisingly perhaps given its crudeness, and included to provide an example calculation. We note however, that this (or any other approximation to *Z_αα′_*) allows the approximate calculation of transition probabilities between states, e.g. extinction probabilities. Such transition probabilities often occur in likelihood-based inference for this kind of dynamical system, and are not accessible through moment-closure type techniques.

Finally, we note that the methods developed above for handling and approximating the dynamics of stochastic spatial models are in no way restricted to population biology, but would also be useful for other systems of reaction-diffusion type.

## Supporting Information

Figure S1A comparison of approximations to the non-spatial SIR model with initial conditions (*S,I,R*) = (480,20,0) and common parameters 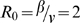 and 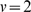. The error-bars denote standard errors from 500 replicates; ‘Taylor’ denotes the approximation developed in Section 3 of Results; and ‘moment closure’ the result from zero third cumulant closure.(0.25 MB EPS)

Box S1 (0.03 MB DOC)

Box S2 (0.03 MB DOC)

Box S3 (0.02 MB DOC)

Box S4 (0.02 MB DOC)

Box S5 (0.02 MB DOC)

Supplementary Material S1 Extra details of calculations, proofs, notation.
(0.19 MB PDF)
