# Supplementary material for: A Many-Body Field Theory Approach to Stochastic Models in Population Biology
Source: PLoS One. 2009 Sep 1;4(9):e6855. doi: 10.1371/journal.pone.0006855 (PMC2734401; doi:10.1371/journal.pone.0006855)
Supplement: Box S1 — (0.03 MB DOC) [file pone.0006855.s002.doc]

**Creation and annihilation operators**

These operators are defined to satisfy the commutation relation

Given a reference (ground-) state |0〉, satisfying *a*|0〉=0, one can define a ‘ladder’ of states by

Obviously, . Because *a* and satisfy the same commutation relation as *x* and *d*/*dx*, and since |0〉 is ‘annihilated’ by the action of *a*,

*a*|*n*〉=*n*|*n*−1〉

Thus creates objects counted by these states, whereas *a* destroys them. The operators can be thought of as moving up and down the ladder of orthogonal states, whence their alternative names: ladder operators, and raising/lowering operators. The operator is called the number operator because it returns the count:

A concrete representation is provided by their first use: the Rodrigues representation of the Hermite polynomials as .
