# Supplementary material for: A Many-Body Field Theory Approach to Stochastic Models in Population Biology
Source: PLoS One. 2009 Sep 1;4(9):e6855. doi: 10.1371/journal.pone.0006855 (PMC2734401; doi:10.1371/journal.pone.0006855)
Supplement: Box S2 — (0.03 MB DOC) [file pone.0006855.s003.doc]

**Fock space**

A Fock space is a very ‘large’ vector space which can be thought of as a data-structure for storing the number and locations of any number of indistinguishable objects. Associated with each point *x* in the space, there are annihilation and creation operators and . Operators based at different points in space satisfy the commutation relation

where is the delta function. Thus they generate independent ‘ladder’-spaces to record the number of objects present at each location in physical space.

Probability distributions over possible configurations are represented by weighted superpositions. For example, suppose we have 1 object present with probability , and two objects present with probability ; and suppose that given there is 1 object, the probability density for its position is , and given 2 objects, their joint density is . This probabilistic state would be represented by the vector:

Note that we are adding together the states containing 1 object (‘1-particle sector’) to states containing 2 objects (‘2-particle sector’). Note also that indistinguishability is built in: and commute, meaning that any asymmetric part of does not contribute to the state.

Informally, the full Fock space is built up as the span of all such states, i.e. the direct sum of all the n-particle subspaces.
