# Supplementary material for: A Many-Body Field Theory Approach to Stochastic Models in Population Biology
Source: PLoS One. 2009 Sep 1;4(9):e6855. doi: 10.1371/journal.pone.0006855 (PMC2734401; doi:10.1371/journal.pone.0006855)
Supplement: Box S3 — (0.02 MB DOC) [file pone.0006855.s004.doc]

**Coherent states**

Coherent states, are complex eigenvectors of the annihilation operator: *a*|α〉=α|α〉. The state |0〉 is a special case. For real α, they are proportional to a Poisson distribution over all numbers or objects. They are important because they form a (non-orthogonal) basis; and because an inner product with the state |〉 results in a sum over all numbers states which can be used to determine normalizations and expectations of random variables. E.g. the expectation of random variable *X* in a state ψ is

$$\left\langle X\right\rangle_{\psi}=\langle|X|\psi\rangle$$
