# Supplementary material for: A Many-Body Field Theory Approach to Stochastic Models in Population Biology
Source: PLoS One. 2009 Sep 1;4(9):e6855. doi: 10.1371/journal.pone.0006855 (PMC2734401; doi:10.1371/journal.pone.0006855)
Supplement: Box S4 — (0.02 MB DOC) [file pone.0006855.s005.doc]

**Functional derivatives**

A functional is a mapping from a space of functions to the real numbers. Functionals, *F*, are often distinguished from functions, *f*(*x*) by the use of square brackets, so that we write *F*[*f*]. We denote differentiation of functionals by

This can be thought of as analogous to partial differentiation, with *x* indexing the vector *f*(*x*) as *i* might index a vector . Functional differentiation is most often encountered in the calculus of variations. Many dynamical systems have laws which can be derived from variational principles. E.g., a wave equation for a density field ρ(*x*,*t*) can be obtained by varying (using integration by parts):

$$\delta A[\rho]=\int dtdx.\delta\rho(x,t).\frac{\delta}{\delta\rho(x,t)}A[\rho]$$

$$=-2\int dtdx.\delta\rho(x,t).\left(\partial_{t}^{2}\rho-\nabla^{2}\rho\right)$$

For mathematicians, this is the Gâteaux derivative.
