# Supplementary material for: A Many-Body Field Theory Approach to Stochastic Models in Population Biology
Source: PLoS One. 2009 Sep 1;4(9):e6855. doi: 10.1371/journal.pone.0006855 (PMC2734401; doi:10.1371/journal.pone.0006855)
Supplement: Box S5 — (0.02 MB DOC) [file pone.0006855.s006.doc]

**Path integrals**

Path integrals (or functional integrals) are the limit of a many dimensional integral. For a field φ(*x*,*t*), we may consider the Feynman path integral as an integral over all possible field values:

$$\int D[\varphi]\left(...\right)=\int\prod_{x,t} d\varphi(x,t).\left(...\right)$$

where can think of *x* and *t* as continuous indices for the vector φ. Path integrals can often be used to formulate and formally manipulate transition probabilities between configurations. E.g. if a particle, whose position is *x*(*t*), is undergoing Brownian motion subject to a force field −∇*V*(*x*), one can represent probability of the transition from to by

where the path integral sums over all those sums which start at and finish at . In this particular case, the integral can be made rigorous via Wiener integration, and used to prove the famous Feynman-Kac formula. In general, the path integral has not yet been made fully rigorous, and remains a formal tool. Note that the coherent state path integrals used in this paper are subtly different to Feynman integrals in their construction.
