# Supplementary material for: A Many-Body Field Theory Approach to Stochastic Models in Population Biology
Source: PLoS One. 2009 Sep 1;4(9):e6855. doi: 10.1371/journal.pone.0006855 (PMC2734401; doi:10.1371/journal.pone.0006855)
Supplement: Supplementary Material S1 — Extra details of calculations, proofs, notation. (0.19 MB PDF) [file pone.0006855.s007.pdf]

A many-body field theory approach to stochastic models in population biology: supplementary material

P.J. Dodd and N.M. Ferguson

Abstract

The purpose of this document is to provide more details of the calculations, proofs, and fix conventions.

S1 Sketch-proof of moment-hierarchy dynamical law.

In the main text, we mentioned that a generating functional

$$Z[J] = \sum_n \frac{1}{n!} \int d1 \dots dn . J_1 \dots J_n . f^{(n)}(1, \dots, n) \tag{1}$$

of moments

$$f^{(k)}(1, \dots, k) = \langle 1| : n_1 \dots n_k : |v\rangle \tag{2}$$

satisfies the evolution equation

$$\partial_t Z[J] = -H \left[1 + J, \frac{\delta}{\delta J} \right] Z[J] \tag{3}$$

Here we sketch a proof of this.

First note that because the reference state is coherent, we have

$$\langle 1| : f[a^\dagger, a] : |v\rangle = \langle 1| : f[1, a] : |v\rangle \tag{4}$$

Also, it is not difficult to see that commuting an $a^\dagger$ through a's to its left acts like differentiation. In multi-index notation therefore:

$$\langle 1|\underline{a}^{\underline{p}} H(\underline{a}^\dagger, \underline{a})|v\rangle = \langle 1|\underline{a}^{\underline{p}} H \left(1 + \frac{\overleftarrow{\partial}}{\partial \underline{a}}, \underline{a} \right) |v\rangle \tag{5}$$

where the derivative acts to the left. This provides a neat way of calculating the dynamics of a specific normal-ordered moment. In the formal limit, one can replace the partial derivatives with functional derivatives.

Consider further the behaviour of a piece of the hamiltonian which can be represented $\overleftarrow{\underline{\partial}}^{\underline{p}}\underline{a}^{\underline{q}}$. Then

$$\sum_{\underline{n}\geq 0}\frac{1}{\underline{n}!}\underline{J}^{\underline{n}}\langle \underline{a}^{\underline{n}}\overleftarrow{\underline{\partial}}^{\underline{p}}\underline{a}^{\underline{q}}\rangle = \sum_{\underline{n}\geq \underline{p}}\frac{1}{\underline{n}!}\underline{J}^{\underline{n}}\frac{\underline{n}!}{\underline{n-p}!}\langle \underline{a}^{\underline{n-p}}\underline{a}^{\underline{q}}\rangle$$

$$= \underline{J}^{\underline{p}}\sum_{\underline{n}\geq 0}\frac{1}{\underline{n}!}\underline{J}^{\underline{n}}\langle \underline{a}^{\underline{q+n}}\rangle = \underline{J}^{\underline{p}}\left(\frac{\partial}{\partial \underline{J}}\right)^{\underline{q}}\sum_{\underline{n}\geq 0}\frac{1}{\underline{n}!}\underline{J}^{\underline{n}}\langle \underline{a}^{\underline{n}}\rangle \qquad (6)$$

which together with the above, proves the result.

S2 Coherent-state path integrals and the effective action

The material in this section is standard to field theory. It is included here to set conventions and for the benefit of readers who do not have a physics background. For more details, see e.g. Refs.[6,11] of the main text.

Write ϕ for a field where ϕ_α (vaguely discretised) refers to site α. Then take our coherent state definition as

$$|\phi\rangle = e^{\sum_\alpha \phi_\alpha a^\dagger_\alpha}|0\rangle \qquad (7)$$

These states have the properties

$$\begin{aligned} a_\alpha|\phi\rangle &= \phi_\alpha|\phi\rangle \\ \langle\phi|\phi'\rangle &= \exp\left(\sum_\alpha \phi^*_\alpha\phi'_\alpha\right) \\ 1 &= \int\prod_\alpha \frac{d\phi^*_\alpha d\phi_\alpha}{2\pi i}e^{-\sum_\alpha \phi^*_\alpha\phi_\alpha}|\phi\rangle\langle\phi| \\ &= \int\prod_\alpha \frac{d(\mathrm{Re}\phi_\alpha)d(\mathrm{Im}\phi_\alpha)}{\pi}e^{-\sum_\alpha \phi^*_\alpha\phi_\alpha}|\phi\rangle\langle\phi| \end{aligned}$$

S2.1 Coherent state path integral

For illustration, we consider

$$q = \langle\phi_f|a_\beta e^{-Ht}|\phi_i\rangle = \langle\phi_f|a_\beta\prod_k e^{-H\delta t_k}|\phi_i\rangle \qquad (8)$$

where the M δt_k give equally spaced intervals which partition $[0,t]$. Before each $k-$th element in this product, we insert

$$1 = \int\prod_\alpha \frac{d\phi^*_{\alpha,k}d\phi_{\alpha,k}}{N}e^{-\sum_\alpha \phi^*_{\alpha,k}\phi_{\alpha,k}}|\phi_k\rangle\langle\phi_k| \qquad (9)$$

(in slightly abusive notation - dropping subscripts to the αs where we can get away with it). Then

$$q = \int \prod_{k=2}^{M} \left[\frac{1}{N} \prod_{\alpha_k} \int d\phi^*_{\alpha,k} d\phi_{\alpha,k} \right] \int \prod_{\alpha_{M+1}} \frac{d\phi^*_{\alpha,M+1} d\phi_{\alpha,M+1}}{N} e^{-\sum_\alpha \phi^*_{\alpha,M+1}\phi_{\alpha,M+1}} \times$$
$$\int \prod_{\alpha_1} \frac{d\phi^*_{\alpha,1} d\phi_{\alpha,1}}{N} \langle \phi_f | a_\beta | \phi_{M+1} \rangle \langle \phi_1 | \phi_i \rangle \times \prod_{k=1}^{M} e^{-\sum_\alpha \phi^*_{\alpha,k}\phi_{\alpha,k}} \langle \phi_{\alpha,k+1} | e^{-\delta t_k H} | \phi_{\alpha,k} \rangle$$

One then uses

$$e^{\delta t H(a^\dagger, a) = : e^{\delta t H(a^\dagger,a)} : + \mathcal{O}(\delta t^2)} \tag{10}$$

and the properties of coherent states above to obtain (ignoring $\mathcal{O}(\delta t^2)$)

$$q = \int \prod_\alpha \frac{d\phi^*_{\alpha,1} d\phi_{\alpha,M+1}}{N} \frac{d\phi^*_{\alpha,1} d\phi_{\alpha,M+1}}{N} \prod_{k=2}^{M} \left[\prod_{\alpha_k} \frac{1}{N} \int d\phi^*_{\alpha,k} d\phi_{\alpha,k} \right] \times$$
$$\phi_{\beta,M+1} \exp\left(+ \sum_\alpha \left(\phi^*_{\alpha,f} - \phi^*_{\alpha,M+1} \right) \phi_{\alpha,M+1} + \sum_\alpha \phi^*_{\alpha,1} \phi_{\alpha,i} \right) \times$$
$$\exp \sum_\alpha \left(\sum_{k=1}^{M} \left(\phi^*_{\alpha,k+1} - \phi^*_{\alpha,k} \right) \phi_{\alpha,k} - \sum_{k=1}^{M} \delta t_k H_{k+1,k} \right)$$
$$= \int \prod_{k=1}^{M+1} \left[\prod_{\alpha_k} \frac{1}{N} \int d\phi^*_{\alpha,k} d\phi_{\alpha,k} \right] \times$$
$$\phi_{\beta,M+1} \exp \sum_\alpha \left(+\phi^*_{\alpha,1}\phi_{\alpha,i} + \left(\phi^*_{\alpha,1} - \phi^*_{\alpha,i} \right) \phi_{\alpha,i} - \phi^*_{\alpha,1}\phi_{\alpha,i} + |\phi_{\alpha,i}|^2 \right) \times$$
$$\exp \sum_\alpha \left(+ \left(\phi^*_{\alpha,f} - \phi^*_{\alpha,M+1} \right) \phi_{\alpha,M+1} + \sum_{k=1}^{M} \left(\phi^*_{\alpha,k+1} - \phi^*_{\alpha,k} \right) \phi_{\alpha,k} - \sum_{k=1}^{M} \delta t_k H_{k+1,k} \right)$$
$$= \int \prod_{k=1}^{M+1} \left[\prod_{\alpha_k} \frac{1}{N} \int d\phi^*_{\alpha,k} d\phi_{\alpha,k} \right] \phi_{\beta,M+1} \exp \sum_\alpha \left(+\phi^*_{\alpha,i}\phi_{\alpha,i} + \int_0^t \left(\frac{\partial \phi^*_\alpha}{\partial t} \phi_\alpha - H(t) \right) \right)$$
$$= \int \prod_{k=1}^{M+1} \left[\prod_{\alpha_k} \frac{1}{N} \int d\phi^*_{\alpha,k} d\phi_{\alpha,k} \right] \phi_{\beta,M+1} \exp \sum_\alpha \left(+\phi^*_{\alpha,f}\phi_{\alpha,f} - \int_0^t \left(\frac{\partial \phi_\alpha}{\partial t} \phi^*_\alpha + H(t) \right) \right)$$

Taking various 'limits', we write this formally as

$$\langle \phi_f | a_x e^{-Ht} | \phi_i \rangle =$$
$$\int_{\phi(0)=\phi_i}^{\phi^*(t)=\phi^*_f} D[\phi^*\phi] \phi(x,t) e^{\left[\int dy \phi^*(y,t)\phi(y,t) - \int_0^t dt \int dy \left(\phi^* \partial_t \phi + H(\phi^*,\phi) \right) \right]}$$
$$= \int_{\phi(0)=\phi_i}^{\phi^*(t)=\phi^*_f} D[\phi^*\phi] \phi(x,t) e^{\int dy \phi^*(y,t)\phi(y,t) - \tilde{S}[\phi^*,\phi\ ]}$$

and similarly for other 'observables'.

S2.2 Path integral representations of interesting quantities

We willconsider initial states which have poisson-distributed numbers of bugs uniformly in space. This gives a multiple of a coherent state:

$$e^{-\int dx n_0}|n_0\rangle = e^{-\int dx n_0} e^{\int dx n_0 a_x^\dagger}|0\rangle \tag{11}$$

The final state will always be $|1\rangle$ for us, which is coherent. So for this state, e.g.

$$f^{(1)}(x,t) = \int_{\phi(0)=n_0}^{\phi^*(t)=1} D[\phi^*\phi]\phi(x,t)e^{\int dy(\phi^*(t)\phi(t)-n_0)-\tilde{S}[\phi^*,\phi\ ]}$$

$$f^{(2)}(x,y,t) = \int_{\phi(0)=n_0}^{\phi^*(t)=1} D[\phi^*\phi]\phi(x,t)\phi(y,t)e^{\int dy(\phi^*(t)\phi(t)-n_0)-\tilde{S}[\phi^*,\phi\ ]}$$

etc.

More general initial probability distributions can be considered by expanding them in terms of coherent states.

S2.3 The shift trick

In [7], Cardy and Täuber point out one can make a shift $\phi^* = 1+\bar{\phi}$, which changes the term in the exponential to

$$\begin{aligned}
&\int dy \left(\phi^*(t)\phi(t) - n_0 - \int dt\,(\phi^*\partial\phi + H[\phi^*,\phi\ ])\right) \\
&= \int dy \left(1.\phi(t) - n_0 - \int dt\,((\bar{\phi}+1)\partial_t\phi + H[\bar{\phi}+1,\phi\ ])\right) \\
&= \int dy \left(1.\phi(t) - n_0 - \phi(t) + \phi(0) - \int dt\left(\bar{\phi}\partial_t\phi + \tilde{H}[\bar{\phi},\phi\ ]\right)\right) \\
&= \int dy \left(-\int dt\left(\bar{\phi}\partial_t\phi + \tilde{H}[\bar{\phi},\phi\ ]\right)\right) = -S[\bar{\phi},\phi\ ]
\end{aligned}$$

Then we have such things as

$$f^{(1)}(x,t) = \int_{\phi(0)=n_0}^{\bar{\phi}(t)=0} D[\bar{\phi}\phi]\phi(x,t)e^{-S[\bar{\phi},\phi\ ]}$$

$$f^{(2)}(x,y,t) = \int_{\phi(0)=n_0}^{\bar{\phi}(t)=0} D[\bar{\phi}\phi]\phi(x,t)\phi(y,t)e^{-S[\bar{\phi},\phi\ ]}$$

For the bug case then

$$S[\bar{\phi},\phi\ ] = \int dxdt\left[\bar{\phi}\left(\partial_t - \kappa\nabla^2 - \gamma\right)\phi - \lambda\bar{\phi}^2\phi - \int dy V_{xy}(1+\bar{\phi}_y)\phi_y\bar{\phi}_x\phi_x\right]$$

write $\gamma = (\lambda - \mu)$. We will write this more concisely as:

$$S = \bar{\phi}D\phi + \bar{\phi}\phi V\phi - \lambda\bar{\phi}^2\phi + \bar{\phi}\phi V\bar{\phi}\phi \tag{12}$$

with

$$D = \partial_t - \kappa\nabla^2 - \gamma \tag{13}$$

S2.4 Relation of coherent states to probability distributions

A coherent state corresponds to poisson sprinkling in each infinitesimal area. In the translation invariant case, this means a poisson choice of total number sprinkled uniformly across the area. For low densities, this state is a good approximation to a fixed initial number of bugs.

S2.5 Calculation of Γ

Introduce a counting parameter ℓ, which will in fact count the number of loops by its power. From the definitions we have

$$\begin{aligned} e^{(-\Gamma[\phi_m]+J\phi_m)/\ell} &= \int D[\phi]e^{(-S[\phi]+J\phi)/\ell} \\ &= \int D[\phi']e^{(-S[\phi_m]-S_1[\phi_m]\phi'-Q_{\phi_m}[\phi']-R[\phi_m,\phi']+J\phi'+J\phi_m)/\ell} \\ &= e^{(-S(\phi_m)+J\phi_m)/\ell}\int D[\phi]e^{-Q_{\phi_m}[\phi]/\ell+(\Gamma_1-S_1[\phi_m])\phi/\ell-R[\phi_m,\phi]/\ell} \end{aligned}$$

We have substitued for J from its definition as the derivative Γ and shifted the variable of integration. Integer subscripts indicate functional derivatives, Q is the part of the action quadratic in the shifted variable ϕ, and R is the functional taylor series for the shifted action from third order up. Writing $\Delta = \Gamma - S$, this reads

$$\begin{aligned} e^{-\Delta/\ell} &= \int D[\phi]e^{-Q_{\phi_m}[\phi]/\ell+\Delta_1\phi/\ell-R[\phi_m,\phi]/\ell} \\ &= \int D[\phi]e^{-Q_{\phi_m}[\phi]+\Delta_1\phi/\ell^{1/2}-R[\phi_m,\ell^{1/2}\phi]/\ell} \end{aligned} \tag{14}$$

where we have made the rescaling $\phi/\ell^{1/2} \to \phi$. (Rescalings of the 'measure' do not concern us because they respresent an irrelevant constant shift in Γ. More careful treatments consider only the ratio of such integrals as meaningful: see, e.g. [11].) This yields an equations which we can solve iteratively for Γ, order by order in ℓ:

$$\Gamma = \sum_{n=0} \ell^n \Gamma^{(n)} \tag{15}$$

Although it appears that this series contains non-integer powers of ℓ, this is not the case because only even moments of a gaussian integral are not zero.

The lowest term in this series reads:

$$\begin{aligned}\Gamma[\phi_m] &= S[\phi_m] - \log \int D[\phi] e^{-Q_{\phi_m}[\phi]} \\ &= S[\phi_m] + \frac{1}{2} \log \mathrm{Det} S_2[\phi_m] = S[\phi_m] + \frac{1}{2} \mathrm{Tr\ Log} S_2[\phi_m]\end{aligned}$$

where capitals have been used for functional determinants and traces and we have dropped irrelevant constants.

S3 The general multimode fluctuation integral

This section extends the mutlimode fluctuation integral to non-hermitian hamiltonians with sources; a case which does not appear to have been treated previously. It is this case which is needed for effective action calculations for non-quantum situations, such as those we treat. We closely follow the techniques of [14].

S3.1 The action

We are interested in the coherent state path integral integral

$$I = \int D[a\bar{a}] e^{-Q} \tag{16}$$

where the integral has zero limits and

$$Q = \int d\tau \left(\bar{a}^T \partial_\tau a - \bar{a}^T \omega a - a^T f a - \bar{a}^T g \bar{a} - J^T a - K^T \bar{a} \right) \tag{17}$$

The discretized version of the action is (indices refering to time-slices)

$$\begin{aligned}&\sum_{j=1}^{n+1} \left[\bar{a}_j^T (a_j - a_{j-1}) - \epsilon (\bar{a}_j^T \omega_{j-1} a_{j-1} - a_{j-1}^T f_{j-1} a_{j-1} + \bar{a}_j^T g_{j-1} \bar{a}_j) \right] \\ &- \epsilon \sum_{j=1}^{n+1} \left[J_j^T a_j + K_j^T \bar{a}_j \right]\end{aligned} \tag{18}$$

with appropriate ϵ. Writing $a = x + iy$, $\bar{a} = x - iy$ and using where convenient that $\xi_0 = \xi_{n+1} = 0$, the action reads

$$\begin{aligned}&\sum_{j=1}^{n} \left[x_j^T x_j + y_j^T y_j - \epsilon (x_j^T g_{j-1} x_j - i y_j g_{j-1} x_j - i x_j^T g_{j-1} y_j - y_j^T g_{j-1} y_j) \right] \\ &- \epsilon \sum_{j=1}^{n} \left[x_j^T f_j x_j + i y_j^T f_j x_j + i x_j^T f_j y_j - y_j^T f_j y_j \right]\end{aligned}$$

$$\begin{aligned}
&\sum_{j=1}^{n}\left[-x_j^T x_{j-1} - i x_j^T y_{j-1} + i y_j^T x_{j-1} - y_j^T y_{j-1}\right] \\
&-\epsilon \sum_{j=1}^{n}\left[x_j^T \omega_{j-1} x_{j-1} + i x_j^T \omega_{j-1} y_{j-1} - i y_j^T \omega_{j-1} x_{j-1} + y_j^T \omega_{j-1} y_{j-1}\right] \\
&-\epsilon \sum_{j=1}^{n+1}\left[J_j^T (x_j + i y_j) + K_j^T (x_j - i y_j)\right] \qquad (19) \\
&= \sum_{j=1}^{n} z_j^T M_j z_j - \sum_{j=1}^{n} z_j^T L_j z_{j-1} - \sum_{j=1}^{n} u_j^T z_j \qquad (20)
\end{aligned}$$

Here, following [14], we have introduced the coordinate system

$$\begin{aligned}
z &= x \otimes \nu_1 + y \otimes \nu_2 \\
\nu_1 &= \begin{pmatrix} 1 \\ 0 \end{pmatrix} \\
\nu_2 &= \begin{pmatrix} 0 \\ 1 \end{pmatrix} \qquad (21)
\end{aligned}$$

where x and y live in $\mathbb{R}^k$ for a k-mode system. Using this, the action can be written compactly as

$$\begin{aligned}
S &= \sum_{j=1}^{n} z_j^T \left[1 \otimes 1 - \epsilon g_{j-1} \otimes \begin{pmatrix} 1 & -i \\ -i & -1 \end{pmatrix} - \epsilon f_j \otimes \begin{pmatrix} 1 & i \\ i & -1 \end{pmatrix}\right] z_j \\
&\quad + \sum_{j=1}^{n} z_j^T \left[1 \otimes \begin{pmatrix} -1 & -i \\ i & -1 \end{pmatrix} - \epsilon \omega_{j-1} \otimes \begin{pmatrix} 1 & i \\ -i & 1 \end{pmatrix}\right] z_{j-1} \\
&\quad \epsilon \sum_{j=1}^{n} z_j^T \left[J_j \otimes \begin{pmatrix} 1 \\ i \end{pmatrix} + K_j \otimes \begin{pmatrix} 1 \\ -i \end{pmatrix}\right] \qquad (22)
\end{aligned}$$

This allows the identification

$$\begin{aligned}
M_j &= 1 - \epsilon g_{j-1} \otimes \Gamma_2 - \epsilon f_j \otimes \Gamma_1 \\
L_j &= (1 + \epsilon \omega_{j-1}) \otimes \Gamma_3 \\
u_j &= \epsilon \left(J_j \otimes \mu_1 + K_j \otimes \mu_2\right) \qquad (23)
\end{aligned}$$

where

$$\begin{aligned}
\Gamma_1 &= \begin{pmatrix} 1 & i \\ i & -1 \end{pmatrix} \\
\Gamma_2 &= \begin{pmatrix} 1 & -i \\ -i & -1 \end{pmatrix} \\
\Gamma_3 &= \begin{pmatrix} 1 & i \\ -i & 1 \end{pmatrix}
\end{aligned}$$

$$\begin{aligned}\mu_1 &= \begin{pmatrix} 1 \\ i \end{pmatrix} \\ \mu_2 &= \begin{pmatrix} 1 \\ -i \end{pmatrix}\end{aligned} \tag{24}$$

S3.2 The integrations

Repeated use of the formula

$$\int d^n a e^{-a^T A a + b^T a} = \frac{\pi^{n/2}}{(\det A)^{1/2}} e^{(1/4) b^T A^{-1} b} \tag{25}$$

allows us to see that I_n, the discretized version of I, is given by

$$I_n = \pi^{kn} \exp\left[\frac{1}{4}\sum_j u_j'^T M_j'^{-1} u_j'\right] \prod_{j=1}^{n} (\det M_j')^{-1/2} \tag{26}$$

where the M_j' are defined recursively by

$$\begin{aligned} M_j' &= M_j - \frac{1}{4} L_j M_{j-1}'^{-1} L_j^T \\ M_1' &= M_1 \end{aligned} \tag{27}$$

$$\begin{aligned} u_j' &= u_j + \frac{1}{2} L_k M_{k-1}'^{-1} u_{k-1}' \\ u_1' &= u_1 \end{aligned} \tag{28}$$

S3.3 Solving the recursions

One shows by induction that

$$M_k' = M_k - X_k \otimes \Gamma_2 \tag{29}$$

$$u_k' = Y_k \otimes \mu_1 + Z_k \otimes \mu_2 \tag{30}$$

The following properties of the gamma's will be used:

$$\begin{aligned} &\Gamma_2\Gamma_1\Gamma_2 = \Gamma_3\Gamma_2\Gamma_3^T = 4\Gamma_2 \\ &\Gamma_1^2 = \Gamma_2^2 = 0 \\ &\Gamma_3\Gamma_1 = \Gamma_3\Gamma_3^T = 0 \\ &\Gamma_1\Gamma_2 = 2\Gamma_3^T \\ &\Gamma_2\Gamma_1 = 2\Gamma_3 \\ &\Gamma_1\Gamma_3 = 2\Gamma_1 \\ &\Gamma_3\Gamma_2 = 2\Gamma_2 \\ &\Gamma_3^2 = 2\Gamma_3 \end{aligned} \tag{31}$$

and

$$\begin{aligned}
\Gamma_1\mu_1 &= \Gamma_2\mu_2 = 0\\
\Gamma_1\mu_2 &= 2\mu_1\\
\Gamma_2\mu_1 &= 2\mu_2\\
\Gamma_3\mu_1 &= \Gamma_2^T\mu_2 = 0\\
\Gamma_3\mu_2 &= 2\mu_2\\
\Gamma_3^T\mu_1 &= 2\mu_1\\
\mu_1^T\mu_1 &= \mu_2^T\mu_2 = 0\\
\mu_1^T\mu_2 &= 2
\end{aligned} \tag{32}$$

S3.4 The X part

We know

$$\begin{aligned}
M_k' &= M_k\\
&-\frac{1}{4}L_k\left[1-(X_{k-1}+\epsilon g_{k-2})\otimes\Gamma_2-\epsilon f_{k-1}\otimes\Gamma_1\right]^{-1}L_k^T
\end{aligned} \tag{33}$$

Dropping the subscripts for the moment:

$$\begin{aligned}
&\left[1-(X+\epsilon g)\otimes\Gamma_2-\epsilon f\otimes\Gamma_1\right]^{-1} =\\
&1+\left[(X+\epsilon g)\otimes\Gamma_2+\epsilon f\otimes\Gamma_1\right]+\left[(X+\epsilon g)\otimes\Gamma_2+\epsilon f\otimes\Gamma_1\right]^2\\
&+\left[(X+\epsilon g)\otimes\Gamma_2+\epsilon f\otimes\Gamma_1\right]^3+\ldots
\end{aligned} \tag{34}$$

Note that in the recursion, the L's carry factors of Γ_3 and Γ_3^T, only Γ_1 and Γ_2 terms survive this, and both yield Γ_2. This shows the form given above is correct. We wish to compute the recursion satisfied by X to order ϵ however. It is not hard to see that up to order ϵ we have

$$\begin{aligned}
&\left[1-(X+\epsilon g)\otimes\Gamma_2-\epsilon f\otimes\Gamma_1\right]^{-1} =\\
&1+\left[(X+\epsilon g)\otimes\Gamma_2+\epsilon f\otimes\Gamma_1\right]+\Big[2(X+\epsilon g)\epsilon f\otimes\Gamma_3+2\epsilon f(X+\epsilon g)\otimes\Gamma_3^T\Big]\\
&+\left[\epsilon 2XfX\otimes\Gamma_2\right]
\end{aligned} \tag{35}$$

Hitting these with the L's one finds that:

$$X_k = X_{k-1}+\epsilon\Big(\omega_{k-1}X_{k-1}+X_{k-1}\omega_{k-1}^T+g_{k-2}+4X_{k-1}f_{k-1}X_{k-1}\Big) \tag{36}$$

The continuum version of this is precisely

$$\frac{dX}{dt} = g+\omega X+X\omega^T+4XfX \tag{37}$$

It only remains to calculate

$$\begin{aligned}\det M_l' &= \det(M_l - X_l \otimes \Gamma_2) \\ &= \det\left(1 - (X_k + \epsilon g_{k-1}) \otimes \Gamma_2 - \epsilon \otimes \Gamma_1\right) \end{aligned} \tag{38}$$

Note both Γ_1 and Γ_2 are traceless. Using $\det(1+A) = 1 + \mathrm{tr}A + ((\mathrm{tr}A)^2 - \mathrm{tr}A^2) + \ldots$, and keeping only order ϵ, the above becomes

$$\det(M_l) = 1 - 4\epsilon \mathrm{tr}(X_l F_l) \tag{39}$$

We then have

$$\prod_l (\det M_l')^{-1/2} \to \exp\left[2\epsilon \sum_l \mathrm{tr}(X_l f_l)\right] \to \exp\left[2 \int d\tau . \mathrm{tr}(X_\tau f_\tau)\right] \tag{40}$$

S3.5 The u part

From the above relations, it is clear that the form $u_k' = Y_k \otimes \mu_1 + Z_k \otimes \mu_2$ holds. To order ϵ then:

$$\begin{aligned} u_k' &= u_k + \frac{1}{2} L_k u_{k-1}' + \\ & \frac{1}{2} L_k \left[(X + \epsilon g) \otimes \Gamma_2 + \epsilon f \otimes \Gamma_1\right] u_{k-1}' + \frac{1}{2} L_k \left[2(X + \epsilon g)\epsilon f \otimes \Gamma_3 + 2\epsilon f (X + \epsilon g) \otimes \Gamma_3^T\right] u_{k-1}' \\ & + \frac{1}{2} L_k \left[\epsilon 2 X f X \otimes \Gamma_2\right] u_{k-1}' \\ &= u_k + \frac{1}{2} L_k \left[(Y + 2\epsilon f Z + 4\epsilon f X Y) \otimes \mu_1 + (Z + 2(X + \epsilon g) Y + 4\epsilon X f Z + 4\epsilon X f X Y) \otimes \mu_2\right] \\ &= u_k + (1 + \epsilon\omega)\left(Z + 2(X + \epsilon g) Y + 4\epsilon X f Z + 4\epsilon X f X Y\right) \otimes \mu_2 \\ &= u_k + \left(Z + 2(X + \epsilon g) Y + 4\epsilon X f Z + 4\epsilon X f X Y + \epsilon\omega Z + 2\epsilon\omega X Y\right) \otimes \mu_2 \end{aligned} \tag{41}$$

which means

$$\begin{aligned} Y_k &= \epsilon J_k \\ Z_k &= Z_{k-1} + 2 X_{k-1} Y_{k-1} + \epsilon\left(K + 2gY + 4XfZ + 4XfXY + \omega Z + 2\omega XY\right) \\ &= Z_{k-1} + \epsilon\left(K + 2XJ + 4XfZ + \omega Z\right) + \mathcal{O}(\epsilon^2) \end{aligned} \tag{42}$$

which becomes in the continuous limit:

$$\frac{dZ}{dt} = K + 2XJ + (\omega + 4Xf)Z \tag{43}$$

Up to order ϵ we have

$$\begin{aligned} & u'^T M'^{-1} u' = (\epsilon J \otimes \mu_1 + Z \otimes \mu_2)^T M'^{-1} (\epsilon J \otimes \mu_1 + Z \otimes \mu_2) \\ & = (\epsilon J \otimes \mu_1 + Z \otimes \mu_2)^T \left[(\epsilon J + 2\epsilon f Z) \otimes \mu_1 + (Z + \epsilon K + 2\epsilon XJ + 4\epsilon X f Z) \otimes \mu_2\right] \\ & = 4\epsilon Z^T (J + fZ) \end{aligned} \tag{44}$$

Which means that the u contribution becomes

$$\exp\left[\int d\tau Z^T(\tau)\left(J(\tau)+f(\tau)Z(\tau)\right)\right] \tag{45}$$

S3.6 Summary

We have shown that (up to an irrelevant constant factor):

$$I=\exp\left[\int d\tau\left(Z^T J+Z^T fZ+2.\mathrm{tr}(Xf)\right)\right] \tag{46}$$

where, with trivial initial conditions

$$\begin{aligned}\frac{dX}{dt} &= g+\omega X+X\omega^T+4XfX\\ \frac{dZ}{dt} &= K+2XJ+(\omega+4Xf)Z\end{aligned} \tag{47}$$

In the main text, we have written Y rather than Z to avoid confusion with generating functionals.

S4 Explicit computations for the two models considered

In this section, there are more details of the calculations pertaining to the specific models we consider.

S4.1 Non-spatial bugs

For our action f, g and ω are given by

$$f = -V\bar{\phi}_m - V\bar{\phi}_m^2 \tag{48}$$

$$g = \lambda\phi_m - V\phi_m^2 \tag{49}$$

$$\omega = \gamma - V - 2V\phi_m + 2\lambda\bar{\phi}_m - 4V\bar{\phi}_m\phi_m \tag{50}$$

Varying this yields the zero loop equations plus a correction (note that $\bar{\phi}\equiv 0$ after variation):

$$0L_s = \partial_s\phi_s - (\gamma-V)\phi_s + V\phi_s^2 \tag{51}$$

$$1L_s = 0L_s - 2\partial_{\bar{\phi}}f(\psi_s)X_s - 2\int_0^t dx f_x\frac{\delta X_x}{\delta\bar{\phi}_s} \tag{52}$$

$$= 0L_s - 2\partial_{\bar{\phi}}f(\psi_s)X_s \tag{53}$$

Note that the dynamical equations in $\bar{\phi}$ are satisfied by the trivial solution. Further, $\partial_{\bar{\phi}}f(\psi_s)=-V$ giving us the system

$$\begin{aligned}\partial_t \phi &= (\gamma - V)\phi - V\phi^2 - 2VX && (54)\\ \partial_t X &= (\lambda\phi - V\phi^2) + 2(\gamma - V - 2V\phi)X && (55)\end{aligned}$$

For comparison, the central moment-closed equations read

$$\begin{aligned}n' &= \gamma n - VN_2\\ N_2' &= 2\gamma N_2 + (\lambda+\mu)n + VN_2 - 2VN_3\\ N_3 &= 3nN_2 - 2n^3 && (56)\end{aligned}$$

with the first and second moment (n and N_2) having initial conditions N_0 and $N_0^2 + N_0$ respectively. N_3 is the third moment, posited to satisfy the closure relation in Eqn.56.

S4.2 Non-spatial SIR

From the SIR-action above, one deduces that

$$f = \begin{pmatrix} 0 & \beta(\bar b + \bar b^2 - \bar a - \bar a \bar b)/2 & 0\\ \beta(\bar b + \bar b^2 - \bar a - \bar a\bar b)/2 & 0 & 0\\ 0 & 0 & 0\end{pmatrix} \quad (57)$$

$$g = \begin{pmatrix} 0 & -\beta ab/2 & 0\\ -\beta ab/2 & \beta ab & 0\\ 0 & 0 & 0\end{pmatrix} \quad (58)$$

$$\omega = \begin{pmatrix} -\beta(b + \bar b b) & -\beta(a + a\bar b) & 0\\ \beta(b + 2\bar b b - \bar a b) & \beta(a + 2a\bar b - \bar a a) - \nu & 0\\ 0 & \nu & \end{pmatrix} \quad (59)$$

where the field letters now refer to the mean fields.

The 1-loop effective action is:

$$\Gamma^{(1)} = -2\int d\tau.\mathrm{tr}(fX) \quad (60)$$

After variation, it is clear that the the barred fields are non-dynamical, and stay zero (as they should). Given this, and writing

$$X = \begin{pmatrix} A & B & D\\ B & C & E\\ D & E & F\end{pmatrix} \quad (61)$$

The differential equations for X become:

$$\begin{aligned}\frac{dA}{dt} &= -2\beta bA - 2\beta aB\\ \frac{dB}{dt} &= -\beta ab/2 + \beta bA + (\beta(a-b) - \nu)B - \beta aC\\ \frac{dC}{dt} &= \beta ab + 2\beta bB + 2(\beta a - \nu)C\end{aligned}$$

$$\begin{aligned}
\frac{dD}{dt} &= -\beta bD - \beta aE + \nu B \\
\frac{dE}{dt} &= \beta bD + (\beta a - \nu)E + \nu C \\
\frac{dF}{dt} &= 2\nu E
\end{aligned} \tag{62}$$

coupled to the equations of motion:

$$\begin{aligned}
\frac{da}{dt} &= -\beta aba - 2\beta B \\
\frac{db}{dt} &= \beta ab - \nu b + 2\beta B \\
\frac{dc}{dt} &= \nu b
\end{aligned} \tag{63}$$

Note that only the first three of the equations in A to F are relevant to the correction term $2B$, in the same was that only 3 of the second moments are relevant in moment-closure. For comparison, the zero third central-moment equations are

$$\begin{aligned}
C'_{SI} &= -\beta SI + (\beta(S-I) - \nu)C_{SI} + \beta IV_S - \beta SV_I - \beta C_{SI} \\
V'_I &= \beta SI + \beta C_{SI} + \nu(I - 2V_I) + 2\beta(SV_I + IC_{SI}) \\
V'_S &= \beta SI + \beta C_{SI} - 2\beta(IV_S + SC_{SI})
\end{aligned} \tag{64}$$

with the covariance C_{SI} and variances V_I and V_S satisfying the initial conditions 0, I_0 and S_0 respectively. The covariance plays the role of the correction to the rate equations for S, I and R.

S4.3 Spatial bugs

We want to write the action in terms of its fourier modes. For a field ψ write

$$\begin{aligned}
\psi(x) &= \sum_n e^{2\pi ix.n/L}\psi_n \\
\psi_n &= \frac{1}{L^d}\int dx e^{-2\pi ix.n/L}\psi(x)
\end{aligned} \tag{65}$$

where d is the number of spatial dimensions.

We have

$$\begin{aligned}
&\int dx \left(\bar{\phi}(x)\partial_t\phi(x) - \kappa\bar{\phi}\nabla^2\phi - \gamma\bar{\phi}\phi\right) \\
&= \int dx \sum_{n,n'} \bar{\phi}_n \left[\partial_t\phi_{n'} + \kappa\frac{(2\pi)^2 n'^2}{L^2}\phi_{n'} - \gamma\phi_{n'}\right] e^{2\pi ix.(n+n')/L} \\
&= L^d \sum_n \bar{\phi}_n \left(\partial_t + \kappa\frac{(2\pi)^2 n^2}{L^2} - \gamma\right)\phi_{-n}
\end{aligned} \tag{66}$$

where we have used

$$\int dx e^{2\pi i x.n/L} = L^d \delta_{n,0} \tag{67}$$

Also,

$$\int dxdy\phi(x)V(x-y)\phi(y) = \int dydx \sum_{n_1,n_2,n_3} \phi_{n_1} V_{n_2} \phi_{n_3} e^{2\pi i/L(x.n_1+n_2.(x-y)+n_3.y)}$$
$$= \sum_{n_1,n_2,n_3} \phi_{n_1} V_{n_2} \phi_{n_3} L^d \delta_{n_1,-n_2} L^d \delta_{n_2,n_3} = L^{2d} \sum_n \phi_n V_{-n} \phi_{-n} \tag{68}$$

The quadratic part of the bugs action is (in condensed notation)

$$\begin{aligned}
Q &= \bar{\phi}\left[\partial_t - \kappa\nabla^2 - \gamma\right]\phi + \bar{\phi}_m \phi V \phi + \bar{\phi}\phi V \phi_m + \bar{\phi}\phi_m V \phi \\
&\quad -\lambda\bar{\phi}\bar{\phi}\phi_m - 2\lambda\bar{\phi}_m\bar{\phi}\phi + 2\bar{\phi}\phi V \bar{\phi}_m\phi_m + \bar{\phi}_m\phi V\bar{\phi}_m\phi + \bar{\phi}\phi_m V\bar{\phi}\phi_m + 2\bar{\phi}\phi_m V\bar{\phi}_m\phi \\
&= \sum_n L^d \bar{\phi}_n \left(\partial_t + \kappa\frac{(2\pi)^2 n^2}{L^2} - \gamma\right)\phi_{-n} + \\
&\quad + \sum_n \left[L^{2d}\bar{\phi}_m\phi_n V_{-n}\phi_{-n} + L^d\bar{\phi}_n\phi_{-n}\bar{V}\phi_m + L^{2d}\phi_m\bar{\phi}_n V_{-n}\phi_{-n}\right] \\
&\quad + \sum_n \left[-L^d\lambda\phi_m\bar{\phi}_n\bar{\phi}_{-n} - 2L^d\lambda\bar{\phi}_m\bar{\phi}_n\phi_{-n} + 2L^d\bar{V}\bar{\phi}_m\phi_m\bar{\phi}_n\phi_{-n}\right] \\
&\quad + \sum_n \left[L^{2d}\phi_m^2\bar{\phi}_n V_{-n}\bar{\phi}_{-n} + L^{2d}\bar{\phi}_m^2\phi_n V_{-n}\phi_{-n} + 2L^{2d}\phi_m\bar{\phi}_m\bar{\phi}_n V_{-n}\phi_{-n}\right]
\end{aligned} \tag{69}$$

Where we have introduced

$$\bar{V} = \int dx V(x) = L^d V_0 \tag{70}$$

Assume $X_{-n} = X_n$, equivalent to $X \in \mathbb{R}$. Summarizing, this gives

$$\begin{aligned}
Q &= L^d \sum_n \left[\bar{\phi}_n\partial_t\phi_n - \bar{\phi}_n\omega\phi_n - \phi_n f_n\phi_n - \bar{\phi}_n g_n\bar{\phi}_n\right] \\
\omega_n &= -\kappa\frac{(2\pi)^2 n^2}{L^2} + \gamma - \bar{V}\phi - L^d\phi V_n + 2\lambda\bar{\phi} - 2\bar{V}\bar{\phi}\phi - 2L^d\phi\bar{\phi}V_n \\
f_n &= -L^d\bar{\phi}V_n - L^d\bar{\phi}^2 V_n \\
g_n &= \lambda\phi - L^d\phi^2 V_n
\end{aligned} \tag{71}$$

where we have dropped the m-subscript. The multimode result is unchanged by the L^d prefactor, and each mode is in fact uncoupled from the rest, giving the effective action 1-loop term in the form

$$\begin{aligned}
\Gamma^{(1)} &= -2\sum_n f_n X_n \\
\dot{X}_n &= g_n + 2\omega_n X_n + 4 f_n X_n^2
\end{aligned} \tag{72}$$

Similar arguments apply to the one loop case, which means we need only consider

$$\begin{aligned} \dot{X}_n &= g_n + 2\omega_n X_n \\ &= \lambda\phi - L^d\phi^2 V_n + 2\left(-\kappa\frac{(2\pi)^2 n^2}{L^2} + \gamma - \bar{V}\phi - L^d\phi V_n\right) X_n \end{aligned} \quad (73)$$

S4.3.1 Converting back to real space

Starting with

$$\dot{X}_n = \lambda\phi - L^d\phi^2 V_n + 2\left(-\kappa\frac{(2\pi)^2 n^2}{L^2} + \gamma - \bar{V}\phi - L^d\phi V_n\right) X_n \quad (74)$$

one can write the correction in terms of real-space again. First note that

$$(V * X)_n = \frac{1}{L^d}\int dxdy.V(x-y)X(y)e^{-2\pi i x.n/L}$$
$$= \sum_{n_1,n_2}\frac{1}{L^d}\int dxdy.V_{n_1}X_{n_2}e^{-2\pi i x.n/L}e^{2\pi i y.n_2/L}e^{2\pi i(x-y).n_1/L} = L^d V_n X_n$$

We therefore have

$$\partial_t X(x) = L^d\lambda\phi.\delta(x) - L^d\phi^2 V(x) + 2(\kappa\nabla^2 + \gamma - \bar{V}\phi)X(x) - 2\phi.(V * X)(x)$$

The correction to the tree-level equations of motion for ϕ is then

$$\frac{2}{L^d}\int dx.V(x)X(x) \quad (75)$$

This compares with a moment-closure correxion of the form

$$\int dx.V(x)C(x) \quad (76)$$

where for 3rd-cumulant-zero closure

$$\partial_t C(x) = 2\lambda\phi.\delta(x) - 2\phi^2 V(x) + 2(\kappa\nabla^2 + \gamma - \bar{V}\phi - V(x))C(x) - 2\phi.(V * C)(x)$$

These are the same, with the identification $C = 2X/L^d$.

S4.4 Spatial SIR

The quadratic part of the action is given by

$$Q = \int d\tau dx\left[\bar{a}^T\partial_\tau a - \int dy\left(\bar{a}_x^T\omega_{xy}a + a_x^T f_{xy}a_y + \bar{a}_x^T g_{xy}\bar{a}_y\right)\right] \quad (77)$$

where

$$f_{xy} = \begin{pmatrix} 0 & \beta V_{xy}(\bar{a}\bar{b}+\bar{a}-\bar{b}^2-\bar{b})/2 & 0 \\ \beta V_{xy}(\bar{a}\bar{b}+\bar{a}-\bar{b}^2-\bar{b})/2 & 0 & 0 \\ 0 & 0 & 0 \end{pmatrix} \quad (78)$$

$$g_{xy} = \begin{pmatrix} 0 & \beta V_{xy}(ab)/2 & 0 \\ \beta V_{xy}(ab)/2 & 0 & -\beta V_{xy}(ab) \\ 0 & 0 & 0 \end{pmatrix} \quad (79)$$

$$\omega_{xy} = \begin{pmatrix} (\kappa\nabla^2+\beta\bar{V}(\bar{b}+1)b)\delta_{xy} & \beta V_{xy}a(\bar{b}+1) & 0 \\ \beta V_{xy}b(\bar{a}-\bar{b})-\beta\bar{V}b(1+\bar{b})\delta_{xy} & (\kappa\nabla^2+\nu+\beta\bar{V}(\bar{a}-\bar{b})a)\delta_{xy}-\beta V_{xy}a(1+\bar{b}) & 0 \\ 0 & -\nu\delta_{xy} & \kappa\nabla^2\delta_{xy} \end{pmatrix} \quad (80)$$

In fourier space

$$Q = \int d\tau L^d \sum_n \left[\bar{a}_n^T \partial_\tau a_{-n} - L^d \left(\bar{a}_n^T \omega_{-n} a_{-n} + a_n^T f_{-n} a_{-n} + \bar{a}_n^T g_{-n} \bar{a}_{-n}\right)\right] \quad (81)$$

As above, we will convert all the $-n$ in this equation to simply n.

$$f_n = \begin{pmatrix} 0 & \beta V_n(\bar{a}\bar{b}+\bar{a}-\bar{b}^2-\bar{b})/2 & 0 \\ \beta V_n(\bar{a}\bar{b}+\bar{a}-\bar{b}^2-\bar{b})/2 & 0 & 0 \\ 0 & 0 & 0 \end{pmatrix} \quad (82)$$

$$g_n = \begin{pmatrix} 0 & \beta V_n(ab)/2 & 0 \\ \beta V_n(ab)/2 & -\beta V_n(ab) & 0 \\ 0 & 0 & 0 \end{pmatrix} \quad (83)$$

$$\begin{aligned}
\omega_n &= \begin{pmatrix} \Omega_1 & \Omega_2 & 0 \\ \Omega_3 & \Omega_4 & 0 \\ 0 & \Omega_5 & \Omega_6 \end{pmatrix} \\
\Omega_1 &= \left[-\kappa(2\pi)^2 n^2/L^2 + \beta\bar{V}(\bar{b}+1)b\right]/L^d \\
\Omega_2 &= \beta V_n a(\bar{b}+1) \\
\Omega_3 &= \beta V_n b(\bar{a}-\bar{b}) - \beta\bar{V}b(1+\bar{b})/L^d \\
\Omega_4 &= \left[-\kappa(2\pi)^2 n^2/L^2 + \nu + \beta\bar{V}(\bar{a}-\bar{b})a\right]/L^d - \beta V_n a(1+\bar{b}) \\
\Omega_5 &= -\nu/L^d \\
\Omega_6 &= -\kappa\frac{(2\pi)^2 n^2/L^2}{L^d}
\end{aligned} \quad (84)$$

Again, everything separates out by mode. The one loop correction is of the form $2L^d \sum_n \mathrm{tr} f_n X_n$ with

$$\dot{X}_n = g_n + \omega_n X_n + X\omega_n^T \quad (85)$$

Dropping the ns and writing

$$X = \begin{pmatrix} A & B & D \\ B & C & E \\ D & E & F \end{pmatrix} \quad (86)$$

we have

$$\begin{aligned}
\frac{dA}{dt} &= 2\Omega_1 A + 2\Omega_2 B \\
\frac{dB}{dt} &= L^d \beta V_n (ab)/2 + \Omega_3 A + (\Omega_1 + \Omega_4) B + \Omega_2 C \\
\frac{dC}{dt} &= -L^d \beta V_n (ab) + 2\Omega_3 B + 2\Omega_4 C \\
\frac{dD}{dt} &= \Omega_5 B + (\Omega_1 + \Omega_6) D + \Omega_2 E \\
\frac{dE}{dt} &= \Omega_5 C + \Omega_3 D + (\Omega_4 + \Omega_6) E \\
\frac{dF}{dt} &= 2\Omega_5 E + 2\Omega_6 F
\end{aligned} \tag{87}$$

where for these calculations

$$\begin{aligned}
\Omega_1 &= \left[-\kappa (2\pi)^2 n^2 / L^2 + \beta \bar{V} b\right] \\
\Omega_2 &= \beta V_n a L^d \\
\Omega_3 &= -\beta \bar{V} b \\
\Omega_4 &= \left[-\kappa (2\pi)^2 n^2 / L^2 + \nu\right] - \beta V_n a L^d \\
\Omega_5 &= -\nu \\
\Omega_6 &= -\kappa (2\pi)^2 n^2 / L^2
\end{aligned} \tag{88}$$

The correction antisymmetrically applied to the $\dot{S}$ and $\dot{I}$ equation is then $2L^d \sum_n \beta V_n B_n$.

S4.4.1 Converting back to real space

The real-space equations are

$$\begin{aligned}
\partial_t A &= 2\left[\kappa \nabla^2 - \beta \bar{V} b\right] A(x) - 2\beta a V * B(x) \\
\partial_t B &= -V(x) L^d \beta a b / 2 + \beta \bar{V} b A(x) + \left[2\kappa \nabla^2 - \beta \bar{V} b - \nu\right] B(x) + \beta a V * B(x) - \beta a V * C(x) \\
\partial_t C &= V(x) L^d \beta a b + 2\beta \bar{V} b B(x) + 2\left[\kappa \nabla^2 - \nu\right] C(x) + 2\beta a V * C(x) \\
\partial_t D &= \nu B(x) + \left[2\kappa \nabla^2 - \beta \bar{V} b\right] D(x) - \beta a V * E(x) \\
\partial_t E &= \nu C(x) + \beta \bar{V} b D(x) + \left[2\kappa \nabla^2 - \nu\right] E(x) + \beta a V * E(x) \\
\partial_t F &= 2\nu E(x) + 2\kappa \nabla^2 F(x)
\end{aligned} \tag{89}$$

and the correction is of the form

$$2 \int dx . V(x) B(x) \tag{90}$$

S5 Variational perturbation calculation

S5.1 Generality

Consider a transition probability

$$Z^{\alpha\alpha_0}[0] = p(\alpha t|\alpha_0 0) = \langle\alpha|e^{-Ht}|\alpha_0\rangle \quad (91)$$
$$= \int_{a_0=\alpha_0}^{\bar{a}_t=\bar{\alpha}-1} D[\bar{a}a]e^{-S[\bar{a},a]} \quad (92)$$

We expand the integration variables a and $\bar{a}$ around paths a_0 and $\bar{a}_0$, with the correct initial and final conditions, respectively, to give

$$Z^{\alpha\alpha_0}[0] = e^{-S[\bar{a}_0,a_0]}\int_{\delta a_0=0}^{\delta\bar{a}_t=0} D[\delta\bar{a}\delta a]e^{-S_Q[\delta\bar{a},\delta a]-S_{int}[\delta\bar{a},\delta a]} \quad (93)$$

where $S_Q[\delta\bar{a},\delta a]$ is the quadratic part of the action and $S_{int}[\delta\bar{a},\delta a]$ is everything else. The idea is to insert a counting parameter ϵ in front of S_{int} and expand perturbatively in this:

$$Z^{\alpha\alpha_0}[0] = e^{-S[\bar{a}_0,a_0]}\int_{\delta a_0=0}^{\delta\bar{a}_t=0} D[\delta\bar{a}\delta a]e^{-S_Q[\delta\bar{a},\delta a]-\epsilon S_{int}[\delta\bar{a},\delta a]} \quad (94)$$
$$= e^{-S[\bar{a}_0,a_0]}\langle e^{-\epsilon S_{int}[\delta\bar{a},\delta a]}\rangle_Q \quad (95)$$
$$= e^{-S[\bar{a}_0,a_0]}\langle\left(1-\epsilon.S_{int}+\frac{\epsilon^2}{2}S_{int}^2+\dots\right)\rangle_Q \quad (96)$$

One truncates this at a given order, e.g. 2:

$$Z_2^{\alpha\alpha_0}[0] = e^{-S[\bar{a}_0,a_0]}\langle 1-\epsilon.S_{int}+\frac{\epsilon^2}{2}S_{int}^2\rangle_Q \quad (97)$$

and then asserts a principal of minimal sensitivity (PMS). Namely, that

$$\frac{\delta}{\delta a_0}Z_2^{\alpha\alpha_0}[0] = \frac{\delta}{\delta\bar{a}_0}Z_2^{\alpha\alpha_0}[0] = 0 \quad (98)$$

S5.2 The mean fields

This transition probability is a special case of a generating functional with $J = K = 0$. We can carry out the above procedure for

$$Z^{\alpha\alpha_0}[J,K] = \int_{a_0=\alpha_0}^{\bar{a}_t=\bar{\alpha}-1} D[\bar{a}a]e^{-S[\bar{a},a]+\int d\tau(J^Ta+K^T\bar{a})} \quad (99)$$

more generally. Then we can calculate the mean fields as

$$\langle a_s\rangle = \frac{\delta}{\delta J_s}Z_n^{1,\alpha_0}[0] \quad (100)$$
$$\langle\bar{a}_s\rangle = \frac{\delta}{\delta K_s}Z_n^{1,\alpha_0}[0] \quad (101)$$

for a truncation at order n.

S5.3 Relating to number states

As defined above, we are referring to the transition probability between Poisson distributions:

$$p(\alpha t|\alpha_0 0) = \sum_{n,m} \frac{\alpha^n \alpha_0^m}{n!m!} p(nt|m0) \tag{102}$$

For large n these things are not so different, but one can also use the completeness relation

$$1 = \int \frac{d^2\alpha}{\pi} |\alpha\rangle\langle\alpha| \tag{103}$$

to write

$$|n\rangle = \int \frac{d^2\alpha}{\pi} |\alpha\rangle\langle\alpha|n\rangle = \int \frac{d^2\alpha}{\pi} \frac{e^{-|\alpha|^2/2}\alpha^n}{n!} |\alpha\rangle \tag{104}$$

CHECK!

S5.4 The non-spatial SIR model

The original action is

$$S = \int d\tau \left(\bar{a}^T \partial_\tau a + \nu(\bar{c} - \bar{b})b - \beta(\bar{b} - \bar{a})(1 + \bar{b})ab \right) \tag{105}$$

which means that

$$\begin{aligned} \frac{d}{dt} S_Q &= \bar{a}^T \partial_t a + \nu(\bar{c} - \bar{b})b - \beta[(\bar{b}_0 - \bar{a}_0)(1 + \bar{b}_0)ab + (\bar{b}_0 - \bar{a}_0)\bar{b}(ab_0 + a_0 b)] \\ & \quad -\beta[(\bar{b} - \bar{a})\bar{b}a_0 b_0 + (\bar{b} - \bar{a})(1 + \bar{b}_0)(ab_0 + a_0 b)] \end{aligned} \tag{106}$$

which means that

$$\begin{aligned} f &= \begin{pmatrix} 0 & 0 & 0 \\ 0 & \beta(\bar{b}_0 - \bar{a}_0)(1 + \bar{b}_0) & 0 \\ 0 & 0 & 0 \end{pmatrix} \\ g &= \begin{pmatrix} 0 & -\beta a_0 b_0/2 & 0 \\ -\beta a_0 b_0/2 & \beta a_0 b_0 & 0 \\ 0 & 0 & 0 \end{pmatrix} \\ \omega &= \begin{pmatrix} -\beta(1 + \bar{b}_0)b_0 & -\beta(1 + \bar{b}_0)a_0 & 0 \\ \beta(\bar{b}_0 - \bar{a}_0)b_0 + \beta(1 + \bar{b}_0)b_0 & \nu + \beta(\bar{b}_0 - \bar{a}_0)a_0 + \beta(1 + \bar{b}_0)a_0 & 0 \\ -\nu & 0 & 0 \end{pmatrix} \end{aligned}$$

and

$$\begin{aligned} \frac{d}{dt} S_{int} &= \beta(\bar{a} - \bar{b})\bar{b}(ab_0 + a_0 b) + \beta(\bar{a} - \bar{b})(1 + \bar{b}_0)ab + \beta(\bar{a}_0 - \bar{b}_0)\bar{b}ab \\ & \quad +\beta(\bar{a} - \bar{b})\bar{b}ab \end{aligned} \tag{107}$$

The extra terms in $\langle \ldots \rangle_Q$ are, at order ϵ (using the fact that the expectation of an odd number of fields is zero)

$$\int_0^t d1.\langle\beta(\bar{a}_1-\bar{b}_1)\bar{b}_1a_1b_1\rangle_Q \tag{108}$$

and at order ϵ^2

$$\begin{aligned}
&\beta\int_0^t d1d2.\langle\\
&[b_0.(\bar{a}\bar{b}a-\bar{b}^2a)+a_0(\bar{a}\bar{b}b-\bar{b}^2b)+\bar{a}_0(\bar{b}ab)+\bar{b}_0.(\bar{a}ab-2\bar{b}ab)+1.((\bar{a}-\bar{b})(ab+\bar{b}ab))]_1\\
&\times[b_0.(\bar{a}\bar{b}a-\bar{b}^2a)+a_0(\bar{a}\bar{b}b-\bar{b}^2b)+\bar{a}_0(\bar{b}ab)+\bar{b}_0.(\bar{a}ab-2\bar{b}ab)+1.((\bar{a}-\bar{b})(ab+\bar{b}ab))]_2\\
&\rangle_Q
\end{aligned} \tag{109}$$

The integrand is

$$\begin{aligned}
&b_0(1)b_0(2).\langle(\bar{a}\bar{b}a)_1(\bar{a}\bar{b}a)_2+(\bar{b}^2a)_1(\bar{b}^2a)_2-2(\bar{a}\bar{b}a)_1(\bar{b}^2a)_2\rangle\\
&+b_0(1)a_0(2).\langle(\bar{a}\bar{b}a)_1(\bar{a}\bar{b}b)_2-(\bar{a}\bar{b}a)_1(\bar{b}^2b)_2-(\bar{b}^2a)_1(\bar{a}\bar{b}b)_2+(\bar{b}^2a)_1(\bar{b}^2b)_2\rangle\\
&+b_0(1)\bar{a}_0(2).\langle(\bar{a}\bar{b}a)_1(\bar{b}ab)_2-(\bar{b}^2a)_1(\bar{b}ab)_2\rangle\\
&+b_0(1)\bar{b}_0(2).\langle(\bar{a}\bar{b}a)_1(\bar{a}ab)_2-(\bar{a}\bar{b}a)_1(2\bar{b}ab)_2-(\bar{b}^2a)_1(\bar{a}ab)_2+(\bar{b}^2a)_1(2\bar{b}ab)_2\rangle\\
&+a_0(1)a_0(2).\langle(\bar{a}\bar{b}b)_1(\bar{a}\bar{b}b)_2-2(\bar{a}\bar{b}b)_1(\bar{b}^2b)_2+(\bar{b}^2b)_1(\bar{b}^2b)_2\rangle\\
&+a_0(1)\bar{a}_0(2).\langle(\bar{a}\bar{b}b)_1(\bar{b}ab)_2-(\bar{b}^2b)_1(\bar{b}ab)_2\rangle\\
&+a_0(1)\bar{b}_0(2).\langle(\bar{a}\bar{b}b)_1(\bar{a}ab)_2-(\bar{a}\bar{b}b)_1(2\bar{b}ab)_2-(\bar{b}^2b)_1(\bar{a}ab)_2+(\bar{b}^2b)_1(2\bar{b}ab)_2\rangle\\
&+\bar{a}_0(1)\bar{a}_0(2).\langle(\bar{b}ab)_1(\bar{b}ab)_2\rangle\\
&+\bar{a}_0(1)\bar{b}_0(2).\langle(\bar{b}ab)_1(\bar{a}ab)_2-(\bar{b}ab)_1(2\bar{b}ab)_2\rangle\\
&+\bar{b}_0(1)\bar{b}_0(2).\langle(\bar{a}ab)_1(\bar{a}ab)_2-2(\bar{a}ab)_1(2\bar{b}ab)_2+(2\bar{b}ab)_1(2\bar{b}ab)_2\rangle\\
&+a_0(1).\langle(\bar{a}\bar{b}b-\bar{b}^2b)_1((\bar{a}-\bar{b})(1+\bar{b})ab)_2\rangle\\
&+b_0(1).\langle(\bar{a}\bar{b}a-\bar{b}^2a)_1((\bar{a}-\bar{b})(1+\bar{b})ab)_2\rangle\\
&+\bar{a}_0(1).\langle(\bar{b}ab)_1((\bar{a}-\bar{b})(1+\bar{b})ab)_2\rangle\\
&+\bar{b}_0(1).\langle(\bar{a}ab-2\bar{b}ab)_1((\bar{a}-\bar{b})(1+\bar{b})ab)_2\rangle\\
&+1.\langle((\bar{a}-\bar{b})(1+\bar{b})ab)_1((\bar{a}-\bar{b})(1+\bar{b})ab)_2\rangle
\end{aligned} \tag{110}$$

One thus has

$$Z_2=e^{-S[\bar{a}_0,a_0]}\left[1-k+\frac{\epsilon^2}{2}.\int_0^t d1L(1)^Ta_0(1)+\frac{\epsilon^2}{2}.\int_0^t d1d2.a_0^T(2)q(2,1)a_0(1)\right] \tag{111}$$

The PMS thus becomes (using a_0 for both the barred and unbarred fields)

$$\frac{\delta}{\delta a_0(1)}S[\bar{a}_0,a_0]Z_2=\left(\epsilon^2.L(1)+\epsilon^2\int_0^2 d2.q(1,2)a_0(2)\right)e^{-S[\bar{a}_0,a_0]} \tag{112}$$

In our case

S5.5 Moments

Calculation of the functions $L(t)$ and $q(t,t')$ requires the computation of the moments of $\langle \ldots \rangle_Q$ up to order 8. These can be calculated from the generating functional I, as above:

$$I = \exp\left(\int d\tau Y^T J + Y^T f Y + 2\mathrm{tr}(Xf)\right) \quad (113)$$

$$\dot{Y} = K + 2XJ + (\omega + 4Xf)Y \quad (114)$$

$$\dot{X} = g + \omega X + X\omega^T + 4XfX \quad (115)$$

Henceforth, we will confine ourselves to computation of mean fields as these are simpler. The equations admit a solution with $\bar{a} \equiv 0$. This can be seen as follows. The $\delta S/\delta a$ terms are all at least linear in barred fields, and so the only forcing is from the L and q terms. If the barred fields are zero, then $f \equiv 0$, so that

$$I = \exp\left(\int d\tau Y^T J\right) \quad (116)$$

$$\dot{Y} = K + 2XJ + \omega Y \quad (117)$$

$$\dot{X} = g + \omega X + X\omega^T \quad (118)$$

The first of these implies that

$$\frac{\delta \dot{Y}_t}{\delta K_s} = \delta(t-s) + \omega_t \frac{\delta Y_t}{\delta K_s} \quad (119)$$

$$\Rightarrow \frac{\delta Y_x}{\delta K_s} = U(x,s)\theta(x-s) \quad (120)$$

$$U(x,s) = T\exp\int_s^x d\tau.\omega_\tau \quad (121)$$

and hence that higher derivatives of Y with respect to K are zero This further implies that

$$\left.\frac{\delta^{i+j} I}{\delta J^i \delta K^j}\right|_0 = 0 \quad (i < j) \quad (122)$$

and this leaves no forcing on the barred dynamics, allowing the solution $\bar{a}_0 \equiv 0$. The terms of the integrand Eqn. 110 which are are relevant are:

$$+b_0(1)\bar{a}_0(2).\langle(\bar{a}\bar{b}a)_1(\bar{b}ab)_2 - (\bar{b}^2a)_1(\bar{b}ab)_2\rangle$$
$$+b_0(1)\bar{b}_0(2).\langle(\bar{a}\bar{b}a)_1(\bar{a}ab)_2 - (\bar{a}\bar{b}a)_1(2\bar{b}ab)_2 - (\bar{b}^2a)_1(\bar{a}ab)_2 + (\bar{b}^2a)_1(2\bar{b}ab)_2\rangle$$
$$+a_0(1)\bar{a}_0(2).\langle(\bar{a}\bar{b}b)_1(\bar{b}ab)_2 - (\bar{b}^2b)_1(\bar{b}ab)_2\rangle$$
$$+a_0(1)\bar{b}_0(2).\langle(\bar{a}\bar{b}b)_1(\bar{a}ab)_2 - (\bar{a}\bar{b}b)_1(2\bar{b}ab)_2 - (\bar{b}^2b)_1(\bar{a}ab)_2 + (\bar{b}^2b)_1(2\bar{b}ab)_2\rangle$$
$$+\bar{a}_0(1).\langle(\bar{b}ab)_1((\bar{a}-\bar{b})(1+\bar{b})ab)_2\rangle$$
$$+\bar{b}_0(1).\langle(\bar{a}ab - 2\bar{b}ab)_1((\bar{a}-\bar{b})(1+\bar{b})ab)_2\rangle$$
$$+a_0(1).\langle(\bar{a}\bar{b}b - \bar{b}^2b)_1((\bar{a}-\bar{b})ab)_2\rangle$$

$$+b_0(1).\langle(\bar{a}\bar{b}a-\bar{b}^2a)_1((\bar{a}-\bar{b})ab)_2\rangle$$
$$+1.\langle((\bar{a}-\bar{b})(1+\bar{b})ab)_1((\bar{a}-\bar{b})(1+\bar{b})ab)_2\rangle \tag{123}$$

Since, Wick's theorem applies in this case, all expectations become reduce to products of the form

$$\langle\bar{a}_1a_2\rangle=(*) \tag{124}$$

To reliably compute these without worrying about regularization etc, we retreat to the operator formalism. There,

$$(*a)=\langle 1|U(t,1)(a^\dagger-1)U(1,2)aU(2,0)|0\rangle \tag{125}$$

if $1>2$, and

$$(*b)=\langle 1|U(t,2)aU(2,1)(a^\dagger-1)U(1,0)|0\rangle \tag{126}$$

when $2>1$. Here, $U(t,s)=T\exp-\int_s^t d\tau H_Q(\tau)$, where

$$H_Q(\tau)=(a^\dagger-1)^Tg(a^\dagger-1)+(a^\dagger-1)^T\omega a \tag{127}$$

Defining the Heisenberg operators $q_t=U(t,0)^{-1}qU(t,0)$, we have

$$(*a)=\langle 1|(a^\dagger-1)_1a_2|0\rangle \tag{128}$$
$$(*b)=\langle 1|a_2(a^\dagger-1)_1|0\rangle \tag{129}$$

and we know that

$$\frac{d}{dt}\begin{pmatrix} a_t \\ (a^\dagger-1)_t\end{pmatrix} = U^{-1}\begin{pmatrix} Ha-aH \\ H(a^\dagger-1)-(a^\dagger-1)H\end{pmatrix}U \tag{130}$$
$$= \begin{pmatrix} -\omega_t & -(g_t+g_t^T) \\ 0 & \omega_t^T\end{pmatrix}\begin{pmatrix} a_t \\ (a^\dagger-1)_t\end{pmatrix} \tag{131}$$

So that

$$\begin{pmatrix} a_t \\ (a^\dagger-1)_t\end{pmatrix} = \begin{pmatrix} A_t & B_t \\ 0 & C_t\end{pmatrix}\begin{pmatrix} a \\ (a^\dagger-1)\end{pmatrix} \tag{132}$$

giving

$$(*a)=0 \tag{133}$$
$$(*b)=A_1C_2^T \tag{134}$$

Since

$$\frac{d}{dt}A^{-T}=\omega_tA^{-T} \tag{135}$$

we have $C=A^{-T}$ and therefore

$$(*b) = A_1 A_2^{-1} = T \exp \int_1^2 ds.\omega_s \tag{136}$$

which agrees with Eqns. 120 and 121 with the understanding that $\theta(0) = 0$. One could obtain this result directly, if more delicately, by considering the discretization which underlies the differential equations.
